# Sedimentary Cobalt Protoporphyrin as a Potential Precursor of Prosthetic Heme Group for Bacteria Inhabiting Fossil Organic Matter-Rich Shale Rock

**DOI:** 10.3390/biom11121913

**Published:** 2021-12-20

**Authors:** Robert Stasiuk, Renata Matlakowska

**Affiliations:** Department of Geomicrobiology, Institute of Microbiology, Faculty of Biology, University of Warsaw, 02-096 Warsaw, Poland; r.stasiuk@biol.uw.edu.pl

**Keywords:** shale rock, protoporphyrin, heme

## Abstract

This study hypothesizes that bacteria inhabiting shale rock affect the content of the sedimentary cobalt protoporphyrin present in it and can use it as a precursor for heme synthesis. To verify this hypothesis, we conducted qualitative and quantitative comparative analyses of cobalt protoporphyrin as well as heme, and heme iron in shale rock that were (i) inhabited by bacteria in the field, (ii) treated with bacteria in the laboratory, and with (iii) bacterial culture on synthetic cobalt protoporphyrin. Additionally, we examined the above-mentioned samples for the presence of enzymes involved in the heme biosynthesis and uptake as well as hemoproteins. We found depletion of cobalt protoporphyrin and a much higher heme concentration in the shale rock inhabited by bacteria in the field as well as the shale rock treated with bacteria in the laboratory. Similarly, we observed the accumulation of protoporphyrin in bacterial cells grown on synthetic cobalt protoporphyrin. We detected numerous hemoproteins in metaproteome of bacteria inhabited shale rock in the field and in proteomes of bacteria inhabited shale rock and synthetic cobalt protoporhyrin in the laboratory, but none of them had all the enzymes involved in the heme biosynthesis. However, proteins responsible for heme uptake, ferrochelatase and sirohydrochlorin cobaltochelatase/sirohydrochlorin cobalt-lyase were detected in all studied samples.

## 1. Introduction

Porphyrins occur both in the biosphere and lithosphere. The best-known porphyrins in the biosphere are heme and chlorophyll, which are compounds playing a key role in the metabolism of living organisms. Geoporphyrins or sedimentary porphyrins are found in the lithosphere. These compounds occur in fossil fuels and asphalt as well as in oil shales and associated source rocks where they are found dominantly as metal complexes. The porphyrins of organisms that lived in distant geological periods are the precursors of geoporphyrins and underwent changes during the diagenesis process [1,2,3,4,5]. The studies carried out so far have indicated the potential use of geoporphyrins by microorganisms currently living on earth, although their importance in the bacterial metabolism and the underlying biochemistry are still unknown [6,7,8,9,10,11].

**Figure 1:**
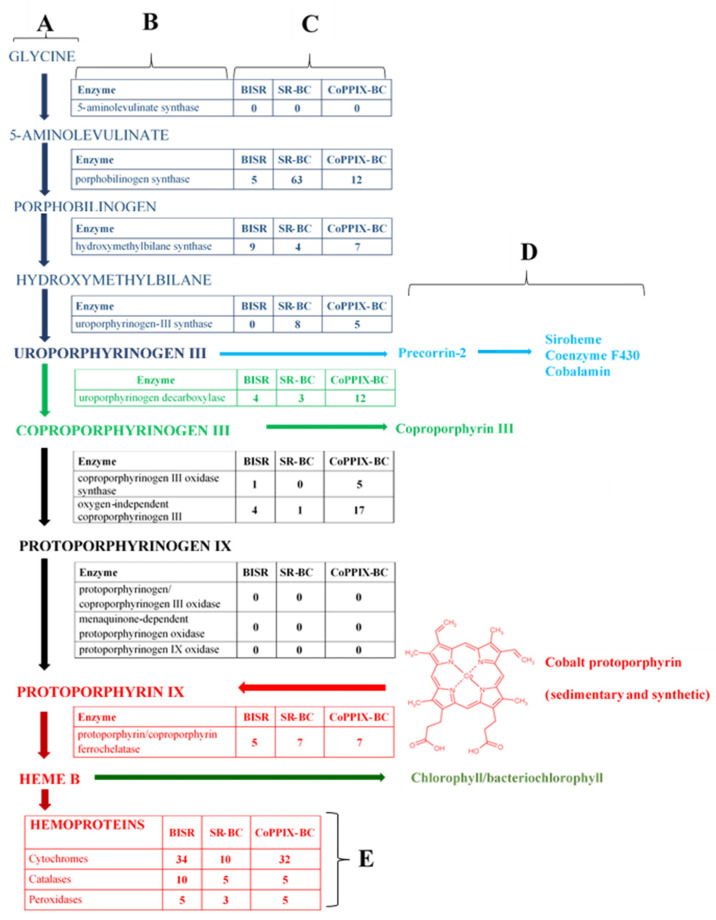
Biosynthesis of heme b including products (**A**) and enzymes (**B**); number of sequences of enzymes involved in heme b biosynthesis detected in metaproteome of BISR (bacteria inhabited shale rock) and proteomes of SR-BC (shale rock-bacterial culture) and CoPPIX-BC (cobalt protoporphyrin-bacterial culture) (**C**); biotransformation of uroporphyrinogen III, coproporphyrinogen and heme b not studied in this study (**D**). Number of sequences of hemoproteins (cytochromes, peroxidases, and catalases) detected in the studied metaproteome of BISRand proteomes of SR-BC and CoPPIX-BC (**E**). The figure does not take into account the transformation of heme b into other types of heme. Numbers of sequences of detected enzymes are presented in Tables S4 and S5. The PPIX transformations studied in this study are marked in red.

One of the most important porphyrins is protoporphyrin IX (PPIX) (C_34_H_34_N_4_O_4_), which plays a crucial role in the metabolism of living organisms as a precursor of critical metalloporphyrins—hemes and chlorophylls. A number of organisms can synthesize PPIX from basic precursors such as glycine and succinyl-CoA or from glutamate, which are converted to aminolevulinic acid (Figure 1A,B) [12]. This compound is then converted to mono-pyrrole (porphobilinogen) and subsequently to tetrapyrrole (porphyrinogen specifically uroporphyrinogen III) and protoporphyrinogen IX, which is oxidized to PPIX by protoporphyrinogen oxidase. Finally, PPIX is converted to heme b (protoheme) (or chlorophyll) by the insertion of iron (or magnesium) cation by enzymes called ferrochelatases (*hemH*; EC: 4.99.1.1; 4.99.1.9) (or magnesium chelatase (EC: 6.6.1.1)). In addition to the protoporphyrin dependent pathway of heme biosynthesis described above, alternative siroheme- and coproporphyrin-dependent pathways of heme biosynthesis are also known [13].

However, numerous bacteria lack some or all the enzymes needed to synthesize their own PPIX and most of them are dependent on exogenous PPIX or require heme acquisition [13,14,15,16,17,18]. These bacteria are diverse in terms of both the demand for the heme group and sources of the heme group used. Usually, they use external sources of heme or its tetrapyrrole precursors. The external heme source is usually blood or the heme-containing enzymes. However, bacteria expressing ferrochelatase can also use tetrapyrroles such as PPIX, coproporphyrinogen, uroporphyrinogen, as well as mesoPPIX, deuteroPPIX, manganese protoporphyrin (MnPPIX), cobalt protoporphyrin (CoPPIX), and zinc protoporphyrin (ZnPPIX) [19,20]. Therefore, the use of tetrapyrrole has been proved. However, it is worth mentioning that bacteria that possess all the enzymes of the heme biosynthetic pathway can also use the external sources of heme [21].

The research presented in this paper is part of a project, the main goal of which is to test whether bacteria inhabiting shale rock rich in fossil organic matter affect sedimentary porphyrins. We conducted our research on the example of subsurface shale rock located in the area of Fore-Sudetic Monocline and known as Kupferschiefer black shale. In this study, we checked if sedimentary CoPPIX may be used/degraded by the bacterial community and the selected bacterial strain. Additionally, weattempted to check if CoPPIX can be used as a source of tetrapyrrole ring for the synthesis of heme b (FePPIX; C_34_H_32_O_4_N_4_Fe) and hemoproteins (cytochromes, catalases and peroxidases) (Figure 1; transformations marked in red). We focused on the protoporphyrin-dependent biosynthetic pathway because, according to the literature, this pathway dominates in bacteria, especially in *Gammapoteobacteria*, which dominate in the studied environment [13,22]. The hypothesis of the study was made based on the fact that CoPPIX is a structural analogue to heme where the iron (II) has been replaced with cobalt (III). The macrocyclic ring structure and the side chain groups are identical. To answer these questions, we divided this study into two parts. In the first part, we analyzed two field samples collected from Kupferschiefer shale rock. The first shale rock (SR) sample was obtained from a freshly exposed rock profile, while the second sample—bacteria-inhabited shale rock (BISR)—was obtained from the profile created 12 years ago. In the second part, SR was exposed to LM27 strain, which was isolated from BISR, for a period of 30 days (SR-BC), and the obtained results were compared with those observed for SR incubated in a sterile mineral medium (sterile control; SR-SC). The results of the analyses were verified in an experiment using synthetic CoPPIX treated with the LM27 strain (bacterial culture; CoPPIX-BC) and incubated in a sterile medium (sterile control; CoPPIX-SC).

As part of the research, we compared: (1) the content of CoPPIX; (2) the content of heme b and heme iron; (3) the presence of hemoproteins such as cytochromes, peroxidase, and catalase; and (4) the presence of enzymes associated with heme biosynthetic pathway; proteins of heme uptake and sirohydrochlorin cobaltochelatase/sirohydrochlorin cobalt-lyase in the metaproteome of BISR and the proteome of the LM27 strain grown on two types of media (SR-BC, CoPPIX-BC). We chose CoPPIX for laboratory tests because literature data suggest that in the studied shale rock, protoporphyrins are present only in complexes with metals, including cobalt [23,24].

## 2. Materials and Methods

### 2.1. Field Samples

BISR and SR were, respectively, collected aseptically from a 12-year-old outcrop and a freshly exposed outcrop (2 days) at a depth of 770 m in Lubin copper mine in Lubińsko-Głogowski Copper District (SW Poland). The samples were obtained from outcrops to a depth of 10 cm. The collected samples were stored at –80 or –4 °C, respectively, until further use. The geochemical characteristics of the samples are presented in our previous publication [22].

### 2.2. Synthetic Porphyrin

A synthetic porphyrin 21H,23H-porphine-2,18-dipropanoic acid, 7,12-diethenyl-3,8,13,17-tetramethyl-cobalt (II)—CoPPIX (C_34_H_32_CoN_4_O_4_Cl; Frontiers Specialty Chemicals, Logan, UT, USA) was used in the laboratory analysis.

### 2.3. Culture Media

Bacteria were grown in lysogeny broth (LB), LB agar [25], or in a modified mineral salts (MBS) medium (prepared by dissolving 800 mg of K_2_HPO_4_ and 200 mg of KH_2_PO_4_·2H_2_O in a liter of demineralized water, pH 7.0 [26]).

### 2.4. Isolation and Identification of Bacteria

The BISR sample (10 g) was resuspended in 20 mL of sterile salt solution (0.85% NaCl). It was shaken at 22 °C for 2 h and streaked onto LB agar. The culture plates were incubated at 22 °C for 1–2 weeks, and single colonies were isolated.

A colony PCR [27] was performed to amplify the 16S rRNA genes. For each bacterial isolate, a single colony was resuspended in 20 µL of PCR lysis solution (50 mM NaOH, 0.25% sodium dodecyl sulfate) and boiled at 95 °C for 15 min. After placing on ice, each sample was mixed with 180 µL of cold, sterile water, and the prepared solutions were used as DNA templates for PCR. The amplification reaction mixture had a volume of 50 µL with the following composition: 1 µL of lysed cell samples, 0.1 nmol of primers (27f and 1492r; [28]), 0.2 mM of dNTPs, and 1 U of Taq polymerase with 1× buffer (Qiagen, Hilden, Germany). PCR was performed in a Mastercycler (Eppendorf, Wesseling, Germany) under the following conditions: initial denaturation at 94 °C for 5 min, and then 20 cycles of 94 °C for 30 s, 53 °C (−1 °C in every successive cycle) for 30 s, and 72 °C for 1.5 min, followed by 15 cycles of 94 °C for 30 s, 46 °C for 30 s, and 72 °C for 1.5 min, and a final extension at 72 °C for 10 min. The obtained PCR products were examined by agarose gel electrophoresis and purified using a PCR Purification Kit (Qiaquick, Qiagen, Germantown, MD, USA). Using the amplified 16S rRNA gene fragments as templates, DNA sequencing was carried out in an ABI Prism 377 automatic sequencer (Applied Biosystems, Foster City, CA, USA). The following three universal primers were used for sequencing: 27f, 515f, and 1492r [28]. The sequence data thus obtained were compared with the 16S rRNA sequences available in the GenBank database using the BLAST program [29].

### 2.5. Culture of LM27 Strain on SR (SR-BC)

The modified MBS medium (500 mL) was supplemented with 50 g of SR. The SR was used after crushing to increase the surface area for the exposure of factors (bacteria, medium, and oxygen); the fragments had a diameter in the range of 0.125–0.25, 0.25–0.5, 0.5–1.0, and 1.0–2.0 mm. Then, the crushed SR was mixed and pasteurized by heating thrice for 1 h at 100 °C, on 3 successive days. This sterilization method has been previously confirmed to minimize the degradation of fossil organic matter (data not shown).

SR-BC was prepared from the bacteria growing in LB medium during the exponential phase and harvested by centrifugation (8000× *g*, 4 °C, 10 min). The pellet was washed thrice in MBS medium (to remove traces of LB medium), resuspended, and inoculated into the MBS medium. The final number of bacterial cells in the inoculated medium was 1 × 10^6^ cells/mL. All experiments were conducted in triplicate. The cultures were grown under static aerobic conditions at 22 °C for 30 days in darkness. The sterile MBS medium supplemented with SR was used as the chemical control (SR-SC).

Bacterial growth was estimated once every 5 days. For this, diluted culture samples were plated on solid LB medium, and the number of colony-forming units was determined (CFU/mL).

After 30 days, BCs and SCs were centrifuged (8000× *g*, 4 °C, 10 min), and the cell-free aqueous phase (supernatant) and solid sample (sediment) were obtained. The sediments were dried at 60 °C and powdered, while the supernatants were filtered through a Whatman filter paper with a pore size of 0.22 µm.

### 2.6. Culture of LM27 Strain on CoPPIX (CoPPIX-BC)

The modified MBS medium (500 mL) was supplemented with glucose (10 mM) and CoPPIX (0.05 mM). The prepared medium was inoculated and incubated as described in subsection Culture of LM27 strain on SR (SR-BC). The sterile MBS medium supplemented with glucose and CoPPIX was used as the chemical control (CoPPIX-SC). All experiments were conducted in triplicate.

### 2.7. Extraction of Organic Matter from the Aqueous Phase (Supernatants)

Organic matter was extracted from 100 mL of cell-free aqueous phase using 25 mL of chloroform in a separatory funnel for 3 min. The extraction procedure was repeated thrice. After extraction, the chloroform extracts were pooled and dried with anhydrous Na_2_SO_4_, while the solvent was evaporated under the N_2_ stream. The extraction was performed in triplicate. A blank sample was prepared by following the same procedure.

### 2.8. Extraction of Organic Matter from Solid Samples (Sediments)

Organic matter was extracted using a dichloromethane/methanol mixture (volume ratio of 9:1) for 4 h using a Soxhlet extraction apparatus (Velp, Usmate Velate, Italy). After extraction, the solvent was evaporated under N_2_ stream. The extraction was performed in triplicate. A blank sample was prepared by following the same procedure.

### 2.9. CoPPIX Analysis Using GC with Mass Spectrometry (MS)

The extracted organic compounds were separated using an Agilent 7890A Series Gas Chromatograph interfaced to an Agilent 5973c Network Mass Selective Detector and an Agilent 7683 Series Injector (Agilent Technologies, Santa Clara, CA, USA). A 5 µL sample was injected with a split ratio of 1:5 by 0.3% standard deviation into an HP-5MS column (30 m × 0.25 mm, 0.25 µm film thickness; Agilent Technologies, USA) using helium as the carrier gas at 1 mL/min. The ion source was maintained at 250 °C. The GC oven was programmed with a temperature gradient starting at 100 °C (for 3 min), which was gradually increased to 300 °C (for 5 min) at 8 °C/min.

MS was carried out in the electron-impact mode at an ionizing potential of 70 eV by selected ion monitoring (SIM). The presence of CoPPIX-specific *m*/*z* 621 ion was monitored.

The qualitative and quantitative analyses of CoPPIX were performed using synthetic CoPPIX as a standard (Figure S1). Its concentration was determined based on the standard curve (0.08, 0.8, 1.6, 16, and 32 µM (50, 500, 1000, 10,000, and 20,000 µg/L)). A concentration curve was plotted with the peak areas corresponding to the concentration of CoPPIX tested (Figure S2). The analysis was performed in triplicate. Error bars represented the classical standard deviation for 3 replicates. The statistical significance of the obtained results was tested using the Student’s *t*-test.

### 2.10. Heme Extraction

Heme was extracted using the method described by Brown et al. [30]. Briefly, 100 mL of the cell-free aqueous phase was mixed with an equal volume of 5% HCl/acetone and clarified by centrifugation (8000× *g*, 4 °C, 20 min). The resulting supernatant was concentrated to 2 mL.

Solid samples (20 g of shale rock or 2 g of bacterial biomass) were frozen at −20 °C for 12 h. Then, the samples were resuspended in 30 mM Tris buffer (pH 8.0) containing 10 mM EDTA and 20% sucrose, and disrupted by sonication on ice for 15 × 1 min, with 1 min pauses (Sonics Vibracell, Newtown, CT, USA; LABO PLUS, Model CV18 head). Following sonication, heme was extracted using the method described above for the liquid sample. The extraction was performed in triplicate.

### 2.11. Heme Analysis Using High-Performance Liquid Chromatography (HPLC) with Photodiode Array Detector (PAD)

Heme was analyzed with an HPLC Waters Separation module 2695 (Waters Alliance, Milford, MA, USA) equipped with a PAD (2996; Waters Alliance, Milford, MA, USA). The solvents used were 0.1% trifluoroacetic acid (TFA)/H_2_O (buffer A) and 0.1% TFA/CH_3_-CN (buffer B). They were filtered through a 0.22 µm filter and degassed with N_2_ before analysis. The sample was loaded at 0.5 mL/min onto a C18 Nova Pack Waters column (3.9 × 150 mm, 5 µm) in 25% buffer B and 75% buffer A, and resolved using a 1%/min gradient from 55% to 75% buffer B (45–25% buffer A). The separated heme was analyzed at a wavelength of 400 nm.

Qualitative and quantitative analyses were performed using bovine heme b (Sigma Aldrich, Hamburg, Germany) as a standard (Figure S3A,B). Heme concentration was determined based on the standard curve (0.0162, 0.162, 1.62, 16.2, 40.5, and 81.0 µM (0.01, 0.1, 1.0, 10, 25, and 50 mg/L)) (Figure S4). Calibration curves were plotted with the peak areas corresponding to the heme concentrations tested. The analysis was performed in triplicate. Error bars represented the classical standard deviation for 3 replicates. The statistical significance of the obtained results was tested using the Student’s *t*-test.

### 2.12. Heme Iron Analysis Using Gas Chromatography (GC) with Atomic Emission Detector (AED)

Heme iron was separated and analyzed on a GC Agilent 7890A Series system (Agilent Technologies, USA) interfaced to a JAS G2350A AED (JAS, Neu-Isenburg, Germany). GC control was carried out using Chemstation ver. B.04.02. A 5 μL sample was introduced into an HP-5 column (30 m × 0.32 mm, 0.50 μm film thickness; Agilent Technologies, USA), with 5% phenyl polysiloxane and 95% dimethyl polysiloxane used for column filling. The injector and detector temperatures were maintained at 380 °C. Sample split 5:1 with He was used as the carrier gas (1 mL/min). The oven temperature was held at 50 °C for 3 min, ramped at 8 °C/min to 100 °C, and finally at 10 °C/min from 100 to 325 °C. The detector was working at carbon, iron, and nitrogen channels, with hydrogen and oxygen used as reaction gases while N_2_ was used as a reference gas.

Heme iron was analyzed using bovine heme as a standard (Figure S5). Its concentration was determined based on the standard curve (0.00016, 0.00162, 0.0162, 0.162, 1.62, and 16.2 µM (0.0001, 0.001, 0.01, 0.1, 1.0, and 10 mg/L)). Calibration curve was plotted with the peak areas corresponding to the concentrations of heme iron tested (Figure S6). The analysis was performed in triplicate. Error bars represented the classical standard deviation for 3 replicates. The statistical significance of the obtained results was tested using the Student’s *t*-test.

### 2.13. Spectrophotometry

CoPPIX accumulation was analyzed using spectral measurements obtained with a Shimadzu UV-1800 spectrophotometer (Shimadzu Corporation, Kyoto, Japan). Spectra were collected every 10 days in the wavelength range of 300–700 nm. High-performance liquid chromatograms and UV–Vis spectra of CoPPIX (425 nm) are presented on Figure S3C,D.

### 2.14. Cobalt Concentration Analysis Using Atomic Absorption Spectrometry

Cobalt concentration was determined in the studied samples using a Solar M6 spectrometer (TJA Solution, Christchurch, Dorset, United Kingdom) by following the manufacturer’s instructions. The analysis was performed in triplicate. Samples for analysis were prepared by digestion with 69% nitric acid. Error bars represented the classical standard deviation for 3 replicates. The statistical significance of the obtained results was tested using the Student’s *t*-test.

### 2.15. Isolation of Proteins

Proteins were isolated using the modified procedure of Ram et al. [31]. Briefly, 10 g of sample was resuspended in 120 mL of 20 mM Tris–HCl (pH 8). The sample was shaken for 3 min and sonicated on ice up to 10 times for 1 min, with 1 min pauses (Sonics Vibracell; LABO PLUS, Model CV18 head). Then, 100 mL of 0.4 M Na_2_CO_3_ (pH 11) was added to the lysed cell suspension, and the suspension was centrifuged at 6000× *g* for 20 min at 4 °C to remove the unlysed cells and cell membrane fragments. The obtained supernatant was filtered through a filter paper with a pore size of 0.22 µm, and the proteins were precipitated from the solution using trichloroacetic acid at a ratio of 1:10 (*v*/*v*). The mixture was allowed to precipitate overnight at 4 °C and then centrifuged at 20,000× *g* for 10 min at 4 °C. The aqueous phase was discarded, and the protein pellet was resuspended in 0.5 mL of precooled methanol (4 °C) and centrifuged at 20,000× *g* for 10 min at 4 °C. The resulting precipitate was dried and stored at −80 °C. The isolation was performed in triplicate.

### 2.16. Identification of Proteins Using Liquid Chromatography (LC) Coupled to Tandem Mass Spectrometry (MS/MS)

The proteins were identified using a Nano-Acquity (Waters) LC system and Orbitrap Velos mass spectrometer (Thermo Electron Corp., San Jose, CA, USA). The analysis was conducted at the Environmental Laboratory of Mass Spectrometry, Institute of Biophysics and Biochemistry (Polish Academy of Sciences, Warsaw, Poland). The equipment used was sponsored in part by the Centre for Preclinical Research and Technology (CePT), a project co-sponsored by the European Regional Development Fund and Innovative Economy, The National Cohesion Strategy of Poland.

Before the analysis, proteins were subjected to a standard “in-solution digestion” procedure. During this process, the proteins were reduced with 50 mM Tris(2-carboxyethyl)phosphine (for 60 min at 60 °C), alkylated with 200 mM *S*-methyl methanethiosulfonate (45 min at room temperature), and digested overnight with trypsin (Sequencing Grade Modified Trypsin; Promega V5111).

The peptide mixture thus obtained was transferred to an RP-18 precolumn (nanoACQUITY Symmetry^®^ C18; Waters 186003514) using water and 0.1% trifluoroacetic acid as mobile phase and then to a nano-HPLC RP-18 column (nanoACQUITY BEH C18; Waters 186003545) using acetonitrile (ACN) gradient (5–35% ACN in 180 min) in the presence of 0.05% formic acid at a flow rate of 250 µL/min. The column outlet was directly coupled to the ion source of the spectrometer which was working in the regime of data-dependent MS to MS/MS switch. A blank run preceded each analysis to ensure a lack of cross-contamination from previous samples.

The raw data acquired were processed by Mascot Distiller followed by Mascot Search (on-site license; Matrix Science, London, UK) against the National Centre for Biotechnology Information (NCBI) protein database. Peptides with a Mascot score exceeding a threshold value corresponding to <5% expectation value, as calculated by the Mascot procedure, were considered as positively identified.

The raw data acquired were processed by Mascot Distiller followed by Mascot Search (on-site license; Matrix Science, London, UK) against the NCBI protein database. Peptides with a Mascot score exceeding a threshold value corresponding to <5% expectation value, as calculated by the Mascot procedure, were considered as positively identified. Metaproteome (BISR) and proteomes (SR-BC, CoPPIX-BC) analysis was performed as described by Kanehisa et al. [32] using GhostKOALA and Kyoto Encyclopedia of Genes and Genomes (KEGG) mapping service.

## 3. Results

### 3.1. Results of BISR and SR Studies

**Figure 2:**
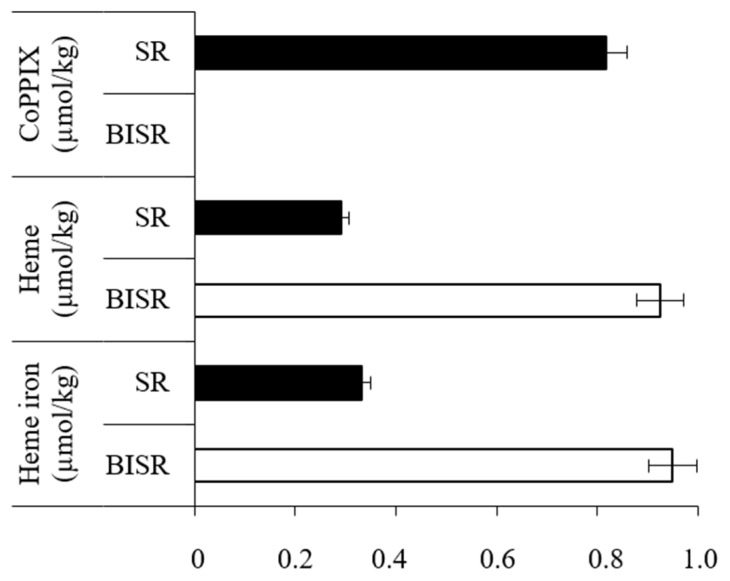
Concentration of CoPPIX, heme and heme iron in BISR and SR samples (µmol/kg of rock sample). Selected ion (*m*/*z* 621) gas chromatogram of CoPPIX and calibration curve for determining the concentration of CoPPIX are presented in Figures S1 and S2, respectively. HPLC-PAD chromatogram of heme is presented in Figure S3A. AED chromatogram of heme is presented in Figure S5. Calibration curves for determining the concentration of heme and heme iron are presented in Figures S4 and S6, respectively. All presented differences are statistically significant (significance level: *p* < 0.05). Supplementary results of chemical analyses of BISR and SR are presented in Figures S7–S12 and Table S1.

As a result of the conducted research, we confirmed lack of CoPPIX-specific *m/z* 621 ion in the BISR sample, while this ion was detected in the SR sample and the CoPPIX concentration was estimated to be 0.817 µmol/kg (Figure 2, Figure S7 and Figure S8; Table S1). At the same time heme was detected in both BISR and SR samples, but its concentration in BISR was found to be over three times higher (0.924 µmol/kg) than in SR (0.291 µmol/kg) (Figure 2 and Figures S3–S5).

Similar differences were found between the studied samples in the content of heme iron (0.948 and 0.332 µmol/kg, respectively) (Figure 2 and Figure S12).

In the BISR metaproteome, we detected 28 sequences matching six of the 11 enzymes involved in heme biosynthesis (Figure 1C and Table S4). These included porphobilinogen synthase, hydroxymethylbilane synthase, uroporphyrinogen decarboxylase, coproporphyrinogen III oxidase, oxygen-independent coproporphyrinogen III oxidase, and protoporphyrin/coproporphyrin ferrochelatase. The sequence numbers of the detected proteins from the NCBI database are given in Table S4. Moreover, eight sequences matching four proteins involved in heme uptake (periplasmic protein TonB, hemoglobin/transferrin/lactoferrin receptor protein (HemR), biopolymer transport protein ExbB, and biopolymer transport protein ExbD) and nine sequences matching sirohydrochlorin cobaltochelatase/sirohydrochlorin cobalt-lyase were identified in BISR (Table S4). In addition to the above, 49 sequences matching hemoproteins were identified in BISR, including 34 sequences of cytochromes, five of peroxidases, and 10 of catalases (Figure 1E; Table S5). In the case of the SR sample, the proteins isolated were not sufficient to perform the above-mentioned analyses.

### 3.2. Isolation and Identification of Pseudomonas spp.

Considering that *Pseudomonas* dominated in the BISR [22], we attempted to isolate the bacteria belonging to this genus. As a consequence, we identified and isolated four *Pseudomonas* strains from the BISR sample. Among them, the LM27 strain was selected for further study. It showed 99% similarity to the species *P. stutzeri* and 100% similarity to the strain LM8 (EU821344) that was previously isolated from shale rock [33].

### 3.3. Results of SR-BC and SR-SC Studies

To verify the results of the analyses on field samples, the LM27 strain was cultivated on the mineral medium with SR in the second part of the study. The growth curve of the LM27 strain cultured on SR mineral medium (SR-BC) is shown in Figure S13.

**Figure 3:**
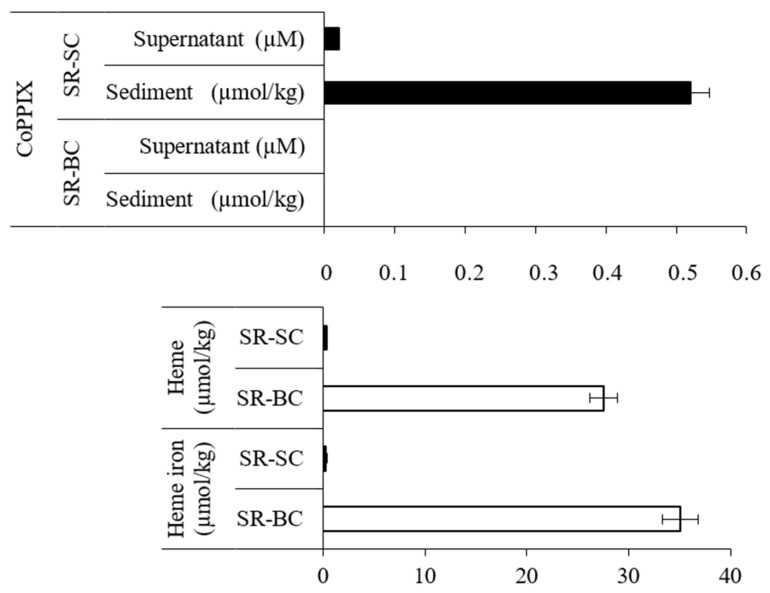
Concentration of CoPPIX, heme and heme iron in SR-BC and SR-SC (µmol/kg of sediment or µM (supernatant)). Selected ion (*m*/*z* 621) gas chromatogram of CoPPIX and calibration curve for determining the concentration of CoPPIX are presented in Figures S1 and S2, respectively. HPLC-PAD chromatogram of heme is presented in Figure S3A. AED chromatogram of heme is presented in Figure S5. Calibration curves for determining the concentration of heme and heme iron are presented in Figures S4 and S6, respectively. All presented differences are statistically significant (significance level: *p* < 0.05). Supplementary results of chemical analyses of SR-BC and SR-SC are presented in Figures S13–S22 and Table S2.

In SR-BC sample no CoPPIX-specific *m*/*z* 621 ion was detected in the supernatant or sediment (Figure 3, Figures S14 and S15 and Table S2). On the other hand, in the SR-SC sample, CoPPIX was detected at a concentration of 0.520 µmol/kg in the sediment and 0.021 µM in the supernatant (Figure 3, Figures S16 and S17). It should be noted that the presence of CoPPIX in the supernatant indicated that protoporphyrin was partially dissolved in the medium. At the same time, heme was detected in the SR-BC sample (Figure 3 and Figures S18–S21), and its content was found to be approximately 80 times greater (27.57 µmol/kg) than in the SR-SC (0.34 µmol/kg) (Figure 3). Similarly, the concentration of heme iron was much higher in the SR-BC sample (35.03 and 0.29 µmol/kg, respectively) (Figure 3 and Figure S22).

In the proteome of the LM27 strain cultivated on SR (SR-BC), we identified 86 sequences that matched six enzymes of the heme biosynthetic pathway (Figure 1B,C and Table S4). Five of enzymes of this pathway were not detected. Seven protein sequences identified in the proteome matched protoporphyrin ferrochelatase; 53 sequences matched four selected proteins involved in heme uptake system and seven matched sirohydrochlorin cobaltochelatase/sirohydrochlorin cobalt-lyase. In addition, 18 sequences matching hemoproteins were identified, including 10 sequences of cytochromes, three of peroxidases, and five of catalases (Figure 1E and Table S5). None of these were detected in SR-SC.

### 3.4. Results of CoPPIX-BC and CoPPIX-SC Studies

In the third part of the study, the LM27 strain cultured on the mineral medium supplemented with synthetic CoPPIX was analyzed. The results of the growth analysis clearly indicated the ability of the strain to grow in the presence of CoPPIX (CoPPIX-BC) (Figure S23).

Accumulation of CoPPIX in the LM27 cells was observed during the growth of the strain on the CoPPIX medium. This phenomenon was monitored by measuring the (i) UV–Vis spectra, (ii) CoPPIX concentration, and (iii) cobalt concentration in the supernatant as well as in the sediment of CoPPIX-BC/SC.

**Figure 4:**
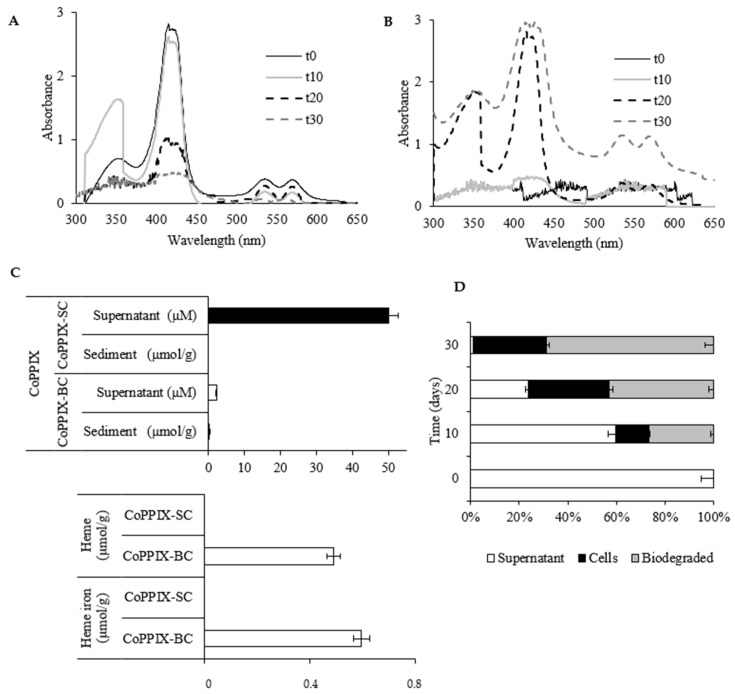
Accumulation of CoPPIX by LM27 strain—UV–Vis spectra of supernatant (**A**) and bacterial cells (**B**); t0–t30: time of the experiment. Concentration of CoPPIX, heme and heme iron in CoPPIX-BC and CoPPIX-SC (µmol/g of biomass or µM (supernatant)) (**C**). Percentage content of CoPPIX in supernatant and cells of CoppIX-BC after 10, 20, and 30 days of cultivation (**D**). Selected ion (*m*/*z* 621) gas chromatogram of CoPPIX and calibration curve for determining the concentration of CoPPIX are presented in Figures S1 and S2, respectively. HPLC-PAD chromatogram of heme is presented in Figure S3A. AED chromatogram of heme is presented in Figure S5. Calibration curves for determining the concentration of heme and heme iron are presented in Figures S4 and S6, respectively. All presented differences are statistically significant (significance level: *p* < 0.05). Supplementary results of chemical analyses of CoPPIX-BC and Co-PPIX-SC are presented in Figures S23–S32 and Table S3.

The UV–Vis spectra (300–700 nm) of CoPPIX obtained for the supernatant and sediment of CoPPIX-BC are shown in Figure 4A,B. The characteristic absorption band of the porphyrin ring was found in the range of 390–425 nm (Soret band), and 10–15 times weaker bands were observed at 480–700 nm (Q-bands) (Figure S3C,D). Soret bands and Q-bands were clearly extinguished in the spectra of CoPPIX-BC supernatant obtained on the 30th day of the experiment (Figure 4A). At the same time, analogous spectra were detected in the cells (sediment) of the LM27 strain (Figure 4B). This finding was accompanied by the gradual disappearance of the characteristic red color of the CoPPIX-BC, and at day 30, the medium was colorless. No such changes in the UV–Vis spectrum or discoloration of the culture medium were observed in the control (CoPPIX-SC) (data not shown).

The values of CoPPIX concentration in CoPPIX-BC confirmed its loss in the supernatant with a simultaneous increase in concentration in the LM27 cells (Figure 4C and Figures S24–S26 and Table S3). Simultaneously, it was found that the concentration of CoPPIX accumulated in the cells decreased during cultivation and reached 0.091 µmol/g of biomass (Figure 4C). On the 30th day of cultivation, the LM27 strain accumulated approximately 90% of CoPPIX, of which almost 70% was used (Figure 4D). In CoPPIX-SC, no such changes in CoPPIX concentration were observed; the concentration of CoPPIX in the supernatant was constant (50 µM) (Figure 4C).

The third parameter that proved the bioaccumulation of CoPPIX was the increase in cobalt concentration in the LM27 cells and its simultaneous decrease in the supernatant (Figure S27). In CoPPIX-SC, no changes in cobalt concentration were observed (data not shown).

In the CoPPIX-BC culture, heme was detected at a concentration of 0.49 µmol/g of biomass and heme iron at 0.59 µmol/g of biomass (Figure 4C, Figures S27 and S32), while no heme was detected in the sterile control (Figure 4C).

In total, 65 sequences of seven enzymes involved in the heme biosynthetic pathway, including seven sequences matching protoporphyrin ferrochelatase, were identified in the proteome of the strain grown on CoPPIX medium (Figure 1B,C; Table S4). In addition, 44 sequences matching four proteins involved in heme uptake and six sequences matching sirohydrochlorin cobaltochelatase/sirohydrochlorin cobalt-lyase were identified (Table S4). Moreover, 42 sequences matching hemoproteins were detected, including 32 sequences matching cytochromes, five matching peroxidases, and five matching catalases (Figure 1E; Table S5). In the control experiments (CoPPIX-SC), no heme-containing proteins were identified.

## 4. Discussion

The experiments on field samples (BISR and SR) and laboratory tests using shale rock (SR-BC/SC) and synthetic CoPPIX (CoPPIX-BC/SC) allowed for a comprehensive analysis of the impact of bacteria on sedimentary CoPPIX. The three parallel analyses, for the first time, clearly demonstrated that sedimentary CoPPIX are affected by bacteria. The absence of CoPPIX in the BISR and SR-BC compared to the SR and SR-SC, respectively, indicates its use by bacteria (Figure 2 and Figure 3). Similarly, the results of the third part of our study showed that the LM27 strain can take up synthetic CoPPIX from the medium, accumulate it in the cell, and then potentially transform (Figure 4C).

Based on these results we can assume that CoPPIX is used by bacteria as a carbon and/or nitrogen source, especially considering that CoPPIX is a water-soluble compound. However, the presence of heme in the BISR, SR-BC, and CoPPIX-BC and the absence of complete set of enzymes involved in biosynthesis of this compound may also indicate the use of CoPPIX as the source of the tetrapyrrole ring. This assumption is also confirmed by the presence of ferrochelatase and hemoproteins in the studied samples (Figure 1). Another important result is the presence of cobalt chelatase (Table S4). According to the literature, this enzyme may also detach cobalt [34]. The systematic name of this enzyme is cobalt-sirohydrochlorin cobalt-lyase (sirohydrochlorin-forming); cobaltochelatase (ambiguous). This result potentially indicates conversion of CoPPIX to PPIX, which then may be chelated with iron by ferrochelatase. It is worth noting that probably the heme biosynthetic pathway enzymes present in the metaproteome of BISR and proteome of SR-BC can be used to synthesize intermediates such as hydroxymethylbilane which can be converted into uroporphyrinogen I or uroporphyrinogen III [35]. Uroporphyrinogen I can be converted into coproporphyrin I and uroporphyrin III. Uroporphyrinogen III is a precursor of precorrin 2, from which siroheme, coenzyme F430 and cobalamin are synthesized (Figure 1D).

The hypothesis that PPIX may be used as a source of tetrapyrrole ring by some bacteria is consistent with previous reports showing that for bacteroids [19], *Lactobacillus lactis* [36], and *Hemophilus influenzae* [37,38]. These bacteria have a shortened heme biosynthetic pathway which involves only ferrochelatase reactions during which an iron atom is inserted in the center of PPIX yielding heme. Similarly, the lack of a complete set of enzymes in heme biosynthetic pathway, with the simultaneous production of this compound, is often observed in bacteria. Especially, it concerns the lack of protoporphyrinogen oxidase. For example, the research by Panek and O’Brian [39] showed that out of 21 examined proteobacterial genomes, 12 were characterized by the lack of the gene encoding protoporphyrinogen oxidase. Additionally, the bioaccumulation of PPIX as well as metal-substituted porphyrins by bacteria has already been described in the literature [40,41,42,43,44]. However, at the same time, we can find reports showing the inhibition of bacterial growth under the influence of certain metalloporphyrins. For example, Stojiljkovic et al. [40], Stojiljkovic and Perkins-Balding [14], and Olczak et al. [45] described the growth inhibition of selected bacteria due to the effect of metalloporphyrins such as gallium(III), cobalt(III), and copper(II) PPIX. Similarly, Fyrestam and Östman [46] showed the effect of additives in the culture medium on the biosynthesis of heme using *Escherichia coli* as a model microorganism. They showed that addition of CoPPIX to the growth medium reduced the amount of heme in *E. coli*, demonstrating this compound’s ability to mimic real heme and inhibit the heme acquisition mechanisms. While Majtan et al. [47] showed that even increased cobalt concentrations in the culture medium of *E. coli* may result in the production of CoPPIX, which was incorporated into heme proteins including membrane-bound cytochromes. The presence of CoPPIX in cytochromes inhibited their electron transport capacity and resulted in a substantially decreased respiration. Finally, the toxicity of heme analogues such as PPIX was explained by Brugna et al. [42]. Using the heme protein catalase in the Gram-positive bacterium *Enterococcus faecalis* as an experimental system, they showed that a variety of heme analogues can be taken up by bacterial cells and incorporated into heme-dependent enzymes. The resulting cofactor-substituted proteins were dysfunctional, generally resulting in arrested cell growth or death. However, it is an important conclusion, that microorganisms that do not rely on exogenous heme for survival such as *E. faecalis*, are resistant to the toxicity of heme analogues. It can be also assumed that the microorganisms to which CoPPIX is toxic do not have the ability to detach cobalt and incorporate iron. Thus, as can be seen from the literature review presented above, the effect of PPIX on bacteria is still unknown. This compound may be toxic, but some studies show that it can also be used by some bacteria.

To sum up, in our research we experimentally proved both the depletion of rock in CoPPIX and the use of synthetic CoPPIX by bacteria. Moreover, the obtained results indicate the potential possibilities of using absolutely unknown sources of the tetrapyrrole ring, i.e., CoPPIX occurring in the lithosphere. However, unambiguous confirmation of this original process is extremely difficult, especially in the case of bacterial communities inhabiting natural environment. All the more difficult in such a complex environment in terms of composition as shale rock. Based on the obtained results, it cannot be excluded that shale rock bacteria may use CoPPIX as a source of carbon and/or nitrogen although it should be noted that in the shale rock more simple compounds are present. Moreover, we cannot exclude that studied bacteria may use other sources of heme. The same may be true for pure cultures. Therefore, it is difficult to apply any control to the CoPPIX experiment (CoPPIX-BC) to confirm that this compound is a source of heme. In our opinion, many compounds can be this source, even heme-containing proteins of dead bacterial cells. It is even more difficult to study this process for shale rock, which is a complex mixture of many different organic compounds. Equally important is the question of what is the significance of other bacteria present in the studied community. Nevertheless, it seems that the obtained results are a prerequisite for undertaking detailed research of this process and looking for a way to clearly confirm it.

The results of this study are critical in understanding the ecology and metabolism of shale-inhabiting bacterial communities. Furthermore, it is extremely important to notice the impact of bacterial metabolism on the geochemistry of shale rocks and other protoporphyrin-containing deposits and reservoir. The described processes also show the continuous transformation of sedimentary rock by microorganisms and also shed new light on the use of porphyrins as biomarkers.

The presented results also indicate an extremely interesting issue, namely the cycle of the tetrapyrrole ring in the environment. Our results show the potential release of the tetrapyrrole ring from fossil organic matter into the biosphere, something completely opposite to what happened during the formation of the sedimentary shale rock. This cycle of tetrapyrrole ring also means mobilization of ancient carbon, associated for millions of years in fossil sedimentary rock, to bacterial biomass and next to global cycle of carbon on the Earth. It is worth remembering that the incorporation of fossil carbon in the circulation may have a crucial impact on global climate [48,49]. In addition, metal transformations must also be taken into account, including both cobalt, which is detached from the organic compound, and iron, which is incorporated into the organic compound [50]. Thus, the described processes are part of the cycle of elements and show the role of the biosphere in these transformations, and at the same time show the potential influence of the lithosphere on the metabolism of the biosphere.

## Data Availability

The data presented in this study are available in supplementary material.

## Supplementary Materials

The following are available online at www.mdpi.com/article/10.3390/biom11121913/s1, Figure S1. SIM chromatogram m/z 621 (A) and mass spectrum of standard CoPPIX (16.1 µM (10 mg/L)) (B), Figure S2. Calibration curve for determining the concentration of CoPPIX using GC–MS; coefficients of determination: R = 0.996067 and R^2^ = 0.992456, Figure S3. High-performance liquid chromatograms and UV–Vis spectra of standards (40.5 µM (25 mg/L))—heme (400 nm) (A,B) and (40.25 µM (25 mg/L)) CoPPIX (425 nm) (C,D)—obtained using HPLC-PAD, Figure S4. Calibration curve for determining the concentration of heme (400 nm) using HPLC-PAD; coefficients of determination: R = 0.994382 and R^2^ = 0.993658, Figure S5. Atomic emission chromatogram of heme (1.62 µM (1 mg/L)) (lines of iron, carbon, and nitrogen were selected) (GC-AED), Figure S6. Calibration curve for determining the concentration of heme iron using GC-AED; coefficients of determination: R = 0.992458 and R^2^ = 0.996365, Figure S7. SIM chromatogram and mass spectrum (inset) of dichloromethane:methanol extract of BISR. SIM chromatogram of CoPPIX is presented in Figure S1 (Supplementary information A), Figure S8. SIM chromatogram and mass spectrum (inset) of dichloromethane:methanol extract of SR. SIM chromatogram of CoPPIX is presented in Figure S1 (Supplementary information A), Figure S9. Detection of heme in BISR and SR samples—HPLC-PAD chromatograms at 400 nm. HPLC-PAD chromatogram of heme is presented in Figure S3A (Supplementary information A), Figure S10. High-performance liquid 3D chromatograms of organic extracts of sediments of BISR and SR obtained using HPLC-PAD, Figure S11. UV–Vis spectra of organic extracts of sediments of BISR and SR obtained using HPLC-PAD, Figure S12. Detection of heme (carbon, nitrogen, and iron) in BISR and SR—GC-AED chromatograms. AED chromatogram of heme is presented in Figure S5 (Supplementary information A), Figure S13. Growth curve of LM27 strain cultured on mineral medium with SR (SR-BC), Figure S14. SIM chromatogram and mass spectrum (inset) of chloroform extract of the supernatant of SR-BC. SIM chromatogram of CoPPIX is presented in Figure S1 (Supplementary information A), Figure S15. SIM chromatogram and mass spectrum (inset) of dichloromethane:methanol extract of the sediment of SR-BC. SIM chromatogram of CoPPIX is presented in Figure S1 (Supplementary information A), Figure S16. SIM chromatogram and mass spectrum (inset) of chloroform extract of the supernatant of SR-SC. SIM chromatogram of CoPPIX is presented in Figure S1 (Supplementary information A), Figure S17. SIM chromatogram and mass spectrum (inset) of dichloromethane:methanol extract of the sediment of SR-SC. SIM chromatogram of CoPPIX is presented in S1 (Supplementary information A), Figure S18. Detection of heme in SR-BC and SR-SC—HPLC-PAD chromatograms at 400 nm. HPLC-PAD chromatogram of heme is presented in Figure S3A (Supplementary information A), Figure S19. High-performance liquid 3D chromatograms of organic extracts of sediments of SR-BC and SR-SC obtained using HPLC-PAD, Figure S20. High-performance liquid 3D chromatograms of organic extracts of supernatants of SR-BC and SR-SC obtained using HPLC-PAD, Figure S21. UV–Vis spectra of organic extracts of sediments of SR-BC and SR-SC obtained using HPLC-PAD, Figure S22. Detection of heme (carbon, nitrogen, and iron) in SR-BC and SR-SC—GC-AED chromatograms. AED chromatogram of heme is presented in Figure S5 (Supplementary information A), Figure S23. Growth curve of LM27 strain cultured on mineral medium with CoPPIX (CoPPIX-BC), Figure S24. SIM chromatogram and mass spectrum (inset) of chloroform extract of the supernatant of CoPPIX-BC. SIM chromatogram of CoPPIX is presented in Figure S1 (Supplementary information A), Figure S25. SIM chromatogram and mass spectrum (inset) of dichloromethane:methanol extract of the sediment of CoPPIX-BC. SIM chromatogram of CoPPIX is presented in Figure S1 (Supplementary information A), Figure S26. SIM chromatogram and mass spectrum (inset) of chloroform extract of the supernatant of CoPPIX-SC. SIM chromatogram of CoPPIX is presented in Figure S1 (Supplementary information A), Figure S27. Accumulation of CoPPIX by LM27 strain: percentage content of cobalt in supernatant and cells of CoPPIX-BC after 10, 20, and 30 days of cultivation, Figure S28. Detection of heme in the sediments of CoPPIX-BC and CoPPIX-SC—HPLC-PAD chromatograms at 400 nm. HPLC-PAD chromatogram of heme is presented in Figure S13A (Supplementary information A), Figure S29. High-performance liquid 3D chromatograms of organic extracts of sediments obtained using HPLC-PAD, Figure S30. High-performance liquid 3D chromatograms of organic extracts of supernatants obtained using HPLC-PAD, Figure S31. UV–Vis spectra of organic extracts of sediments obtained using HPLC-PAD, Figure S32. Detection of heme (carbon, nitrogen, and iron) in CoPPIX-BC and CoPPIX-SC—GC-AED chromatograms. AED chromatogram of heme is presented in Figure S5 (Supplementary information A), Table S1. Concentrations of CoPPIX in BISR and SR based on GC-MS analyses of *m*/*z* 621, Table S2. Concentrations of CoPPIX in SR-BC and SR-SC based on GC-MS analyses of *m*/*z* 621, Table S3. Concentrations of CoPPIX in CoPPIX-BC and CoPPIX-SC based on GC-MS analyses of *m*/*z* 621, Table S4. Numbers of sequences of enzymes involved in heme biosynthesis and uptake and cobalt insertion/disconnection detected in the metaproteome (BISR) and proteomes (SR-BC, CoPPIX-BC); EC—enzyme catalogue, Table S5. Numbers of hemoprotein sequences detected in the metaproteome (BISR) and proteomes (SR-BC, CoPPIX-BC)
