# Supplementary material for: Sedimentary Cobalt Protoporphyrin as a Potential Precursor of Prosthetic Heme Group for Bacteria Inhabiting Fossil Organic Matter-Rich Shale Rock
Source: Biomolecules. 2021 Dec 20;11(12):1913. doi: 10.3390/biom11121913 (PMC8699415; doi:10.3390/biom11121913)
Supplement: Supplementary file 1 [file biomolecules-11-01913-s001.zip › Supplementary information/Supplementary information A. Analyses of standards and calibration curves.pdf]

Supplementary information A. Analyses of standards and calibration curves

A

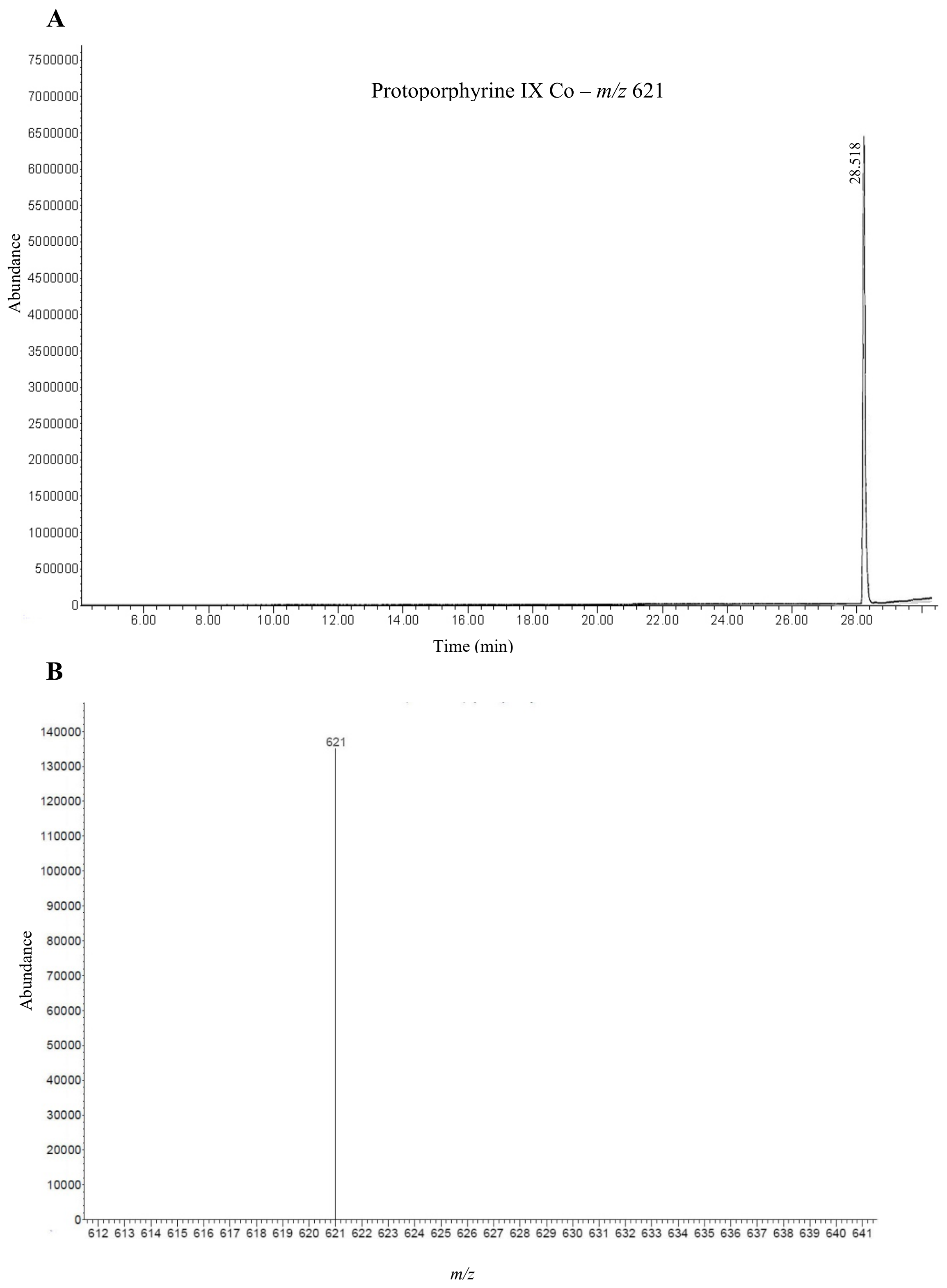

Figure S1. SIM chromatogram *m/z* 621 (A) and mass spectrum of standard CoPPIX (16.1 µM (10 mg/l)) (B)

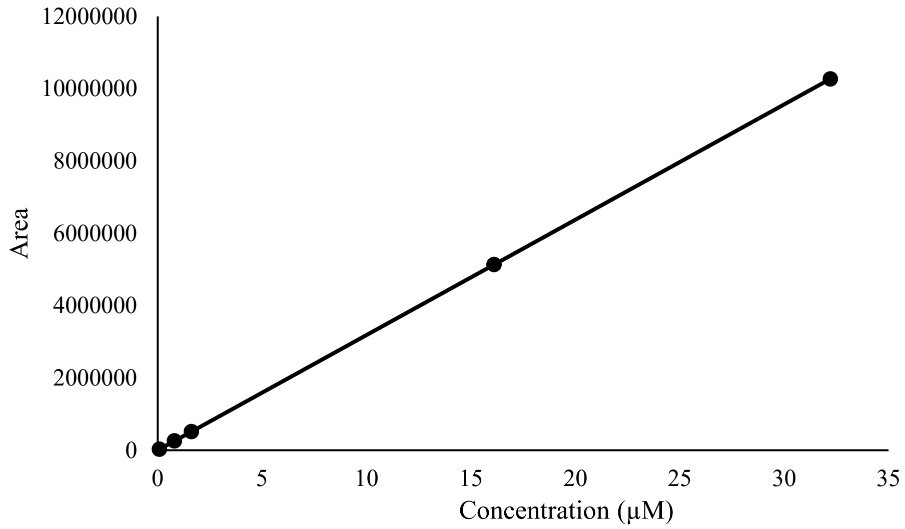

Figure S2. Calibration curve for determining the concentration of CoPPIX using GC–MS; coefficients of determination: R=0.996067 and R^2=0.992456

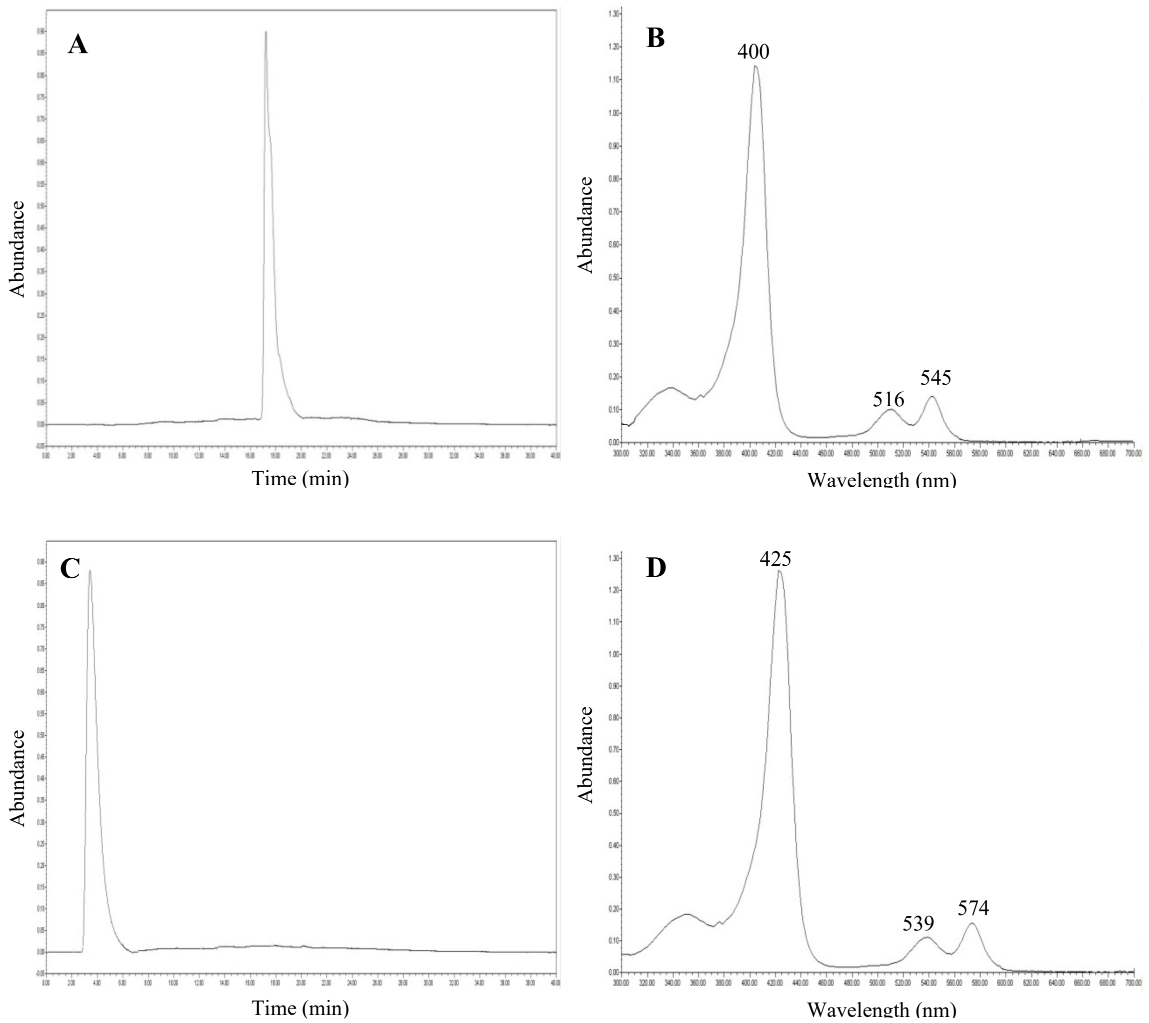

Figure S3. High-performance liquid chromatograms and UV–Vis spectra of standards (40.5 µM (25 mg/l))—heme (400 nm) (A, B) and (40.25 µM (25 mg/l)) CoPPIX (425 nm) (C, D)—obtained using HPLC-PAD

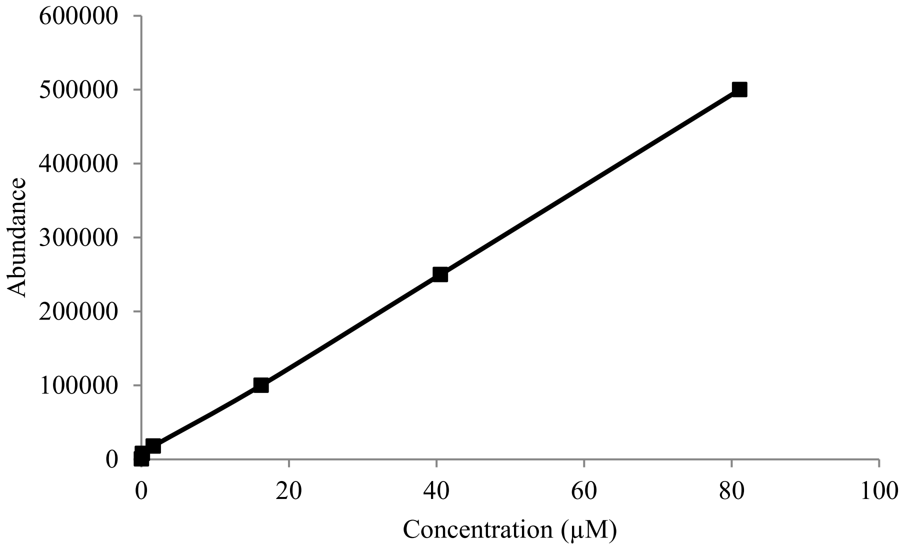

Figure S4. Calibration curve for determining the concentration of heme (400 nm) using HPLC-PAD; coefficients of determination: R=0.994382 and R^2=0.993658

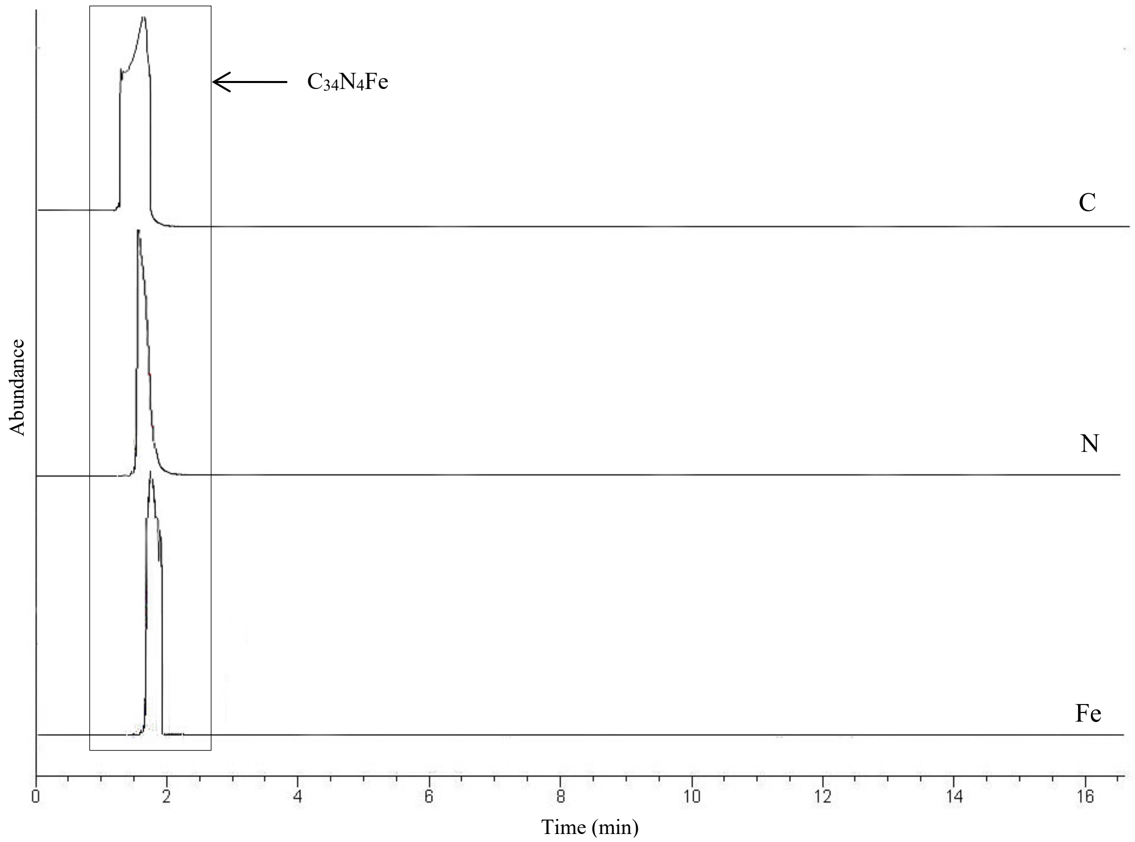

Figure S5. Atomic emission chromatogram of heme (1.62 µM (1 mg/l)) (lines of iron, carbon, and nitrogen were selected) (GC-AED)

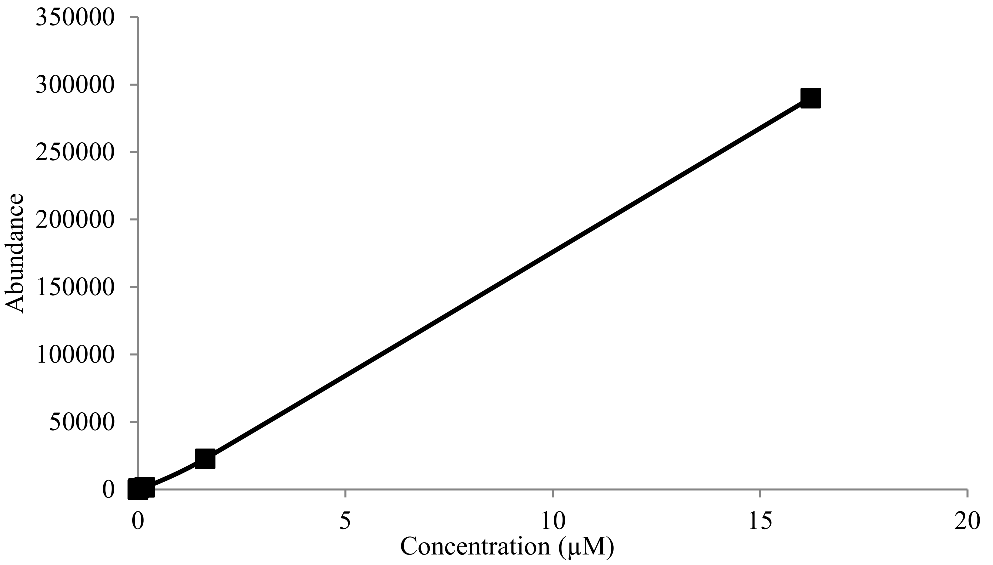

Figure S6. Calibration curve for determining the concentration of heme iron using GC-AED; coefficients of determination: R=0.992458 and R^2=0.996365
