# Supplementary material for: Sedimentary Cobalt Protoporphyrin as a Potential Precursor of Prosthetic Heme Group for Bacteria Inhabiting Fossil Organic Matter-Rich Shale Rock
Source: Biomolecules. 2021 Dec 20;11(12):1913. doi: 10.3390/biom11121913 (PMC8699415; doi:10.3390/biom11121913)
Supplement: Supplementary file 1 [file biomolecules-11-01913-s001.zip › Supplementary information/Supplementary information B. Supplementary results of the chemical analyses of the BISR and SR samples.pdf]

Supplementary information B. Supplementary results of the chemical analyses of the BISR and SR samples

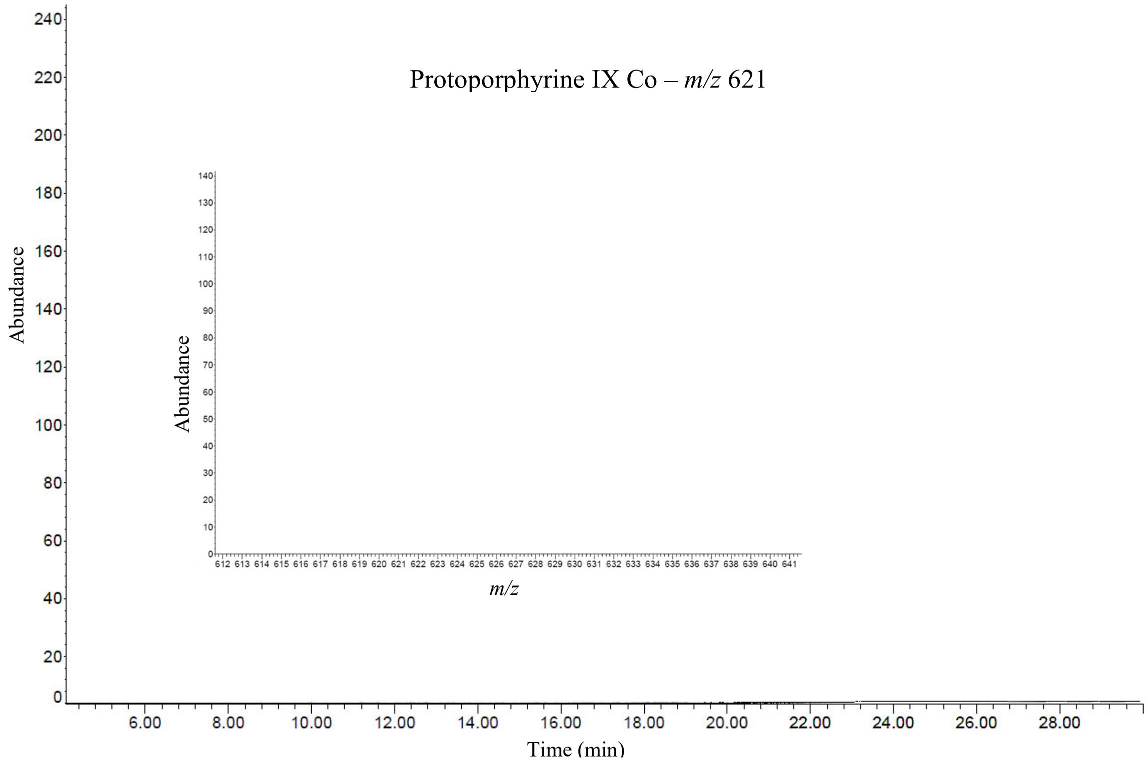

Figure S7. SIM chromatogram and mass spectrum (inset) of dichloromethane:methanol extract of BISR. SIM chromatogram of CoPPIX is presented in Figure S1 (Supplementary information A).

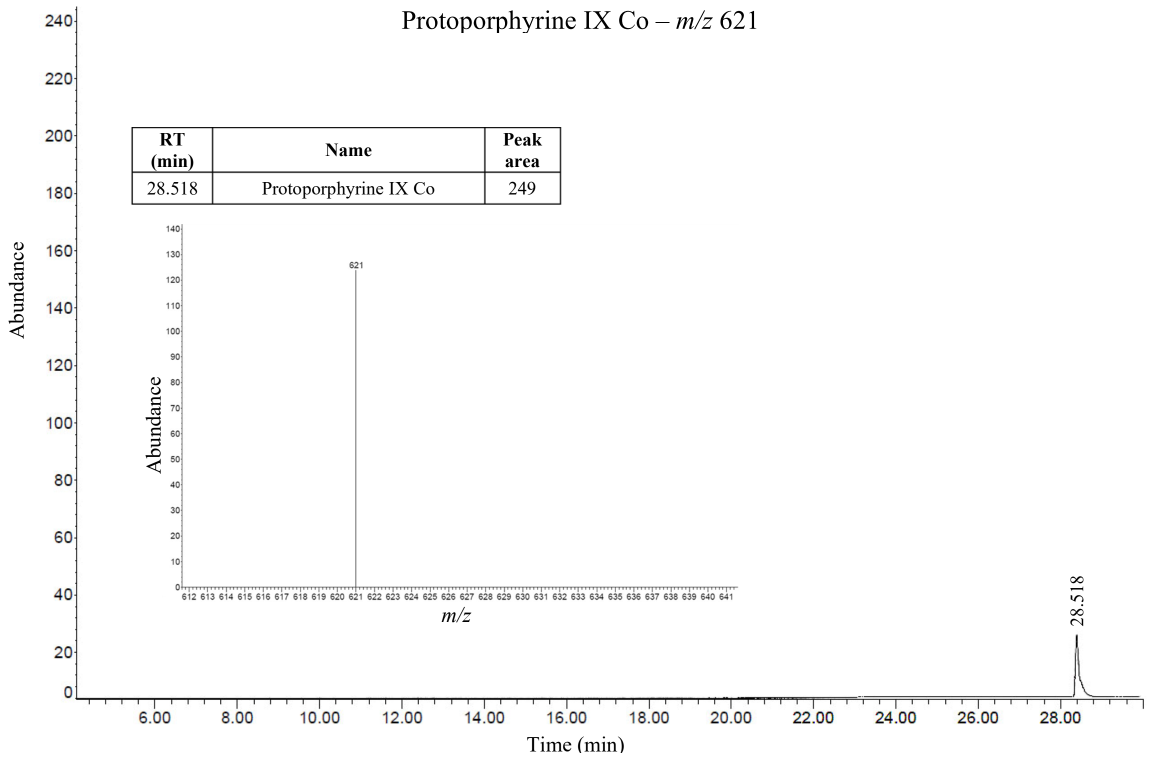

Figure S8. SIM chromatogram and mass spectrum (inset) of dichloromethane:methanol extract of SR. SIM chromatogram of CoPPIX is presented in Figure S1 (Supplementary information A).

.

Table S1. Concentrations of CoPPIX in BISR and SR based on GC-MS analyzes of *m/z* 621

**Sample**	**Sample**			**SD**	**Average**	**Unit**
	**1**	**2**	**3**			
BISR	0	0	0	0	0	µmol/kg
SR	0.824	0.822	0.805	0.010	0.817	µmol/kg

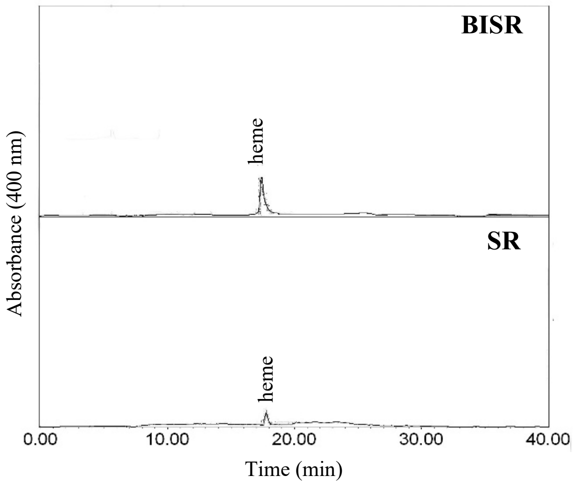

Figure S9. Detection of heme in BISR and SR samples—HPLC-PAD chromatograms at 400 nm. HPLC-PAD chromatogram of heme is presented in Figure S3A (Supplementary information A).

.

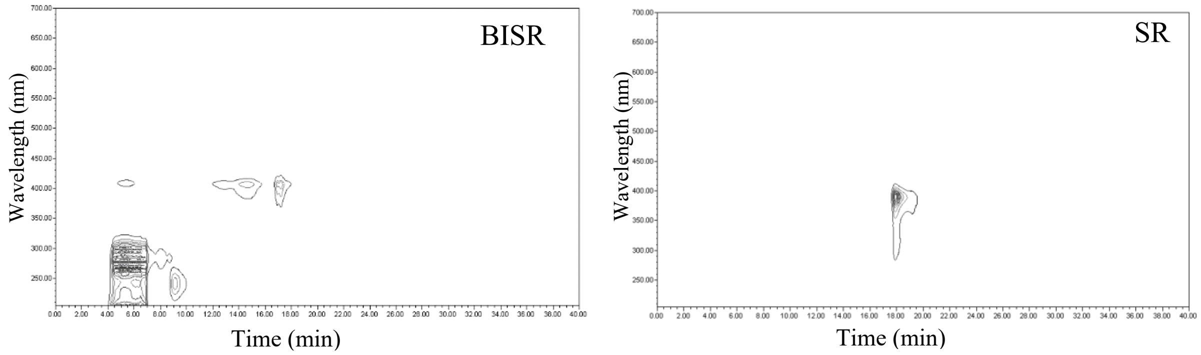

Figure S10. High-performance liquid 3D chromatograms of organic extracts of sediments of BISR and SR obtained using HPLC-PAD

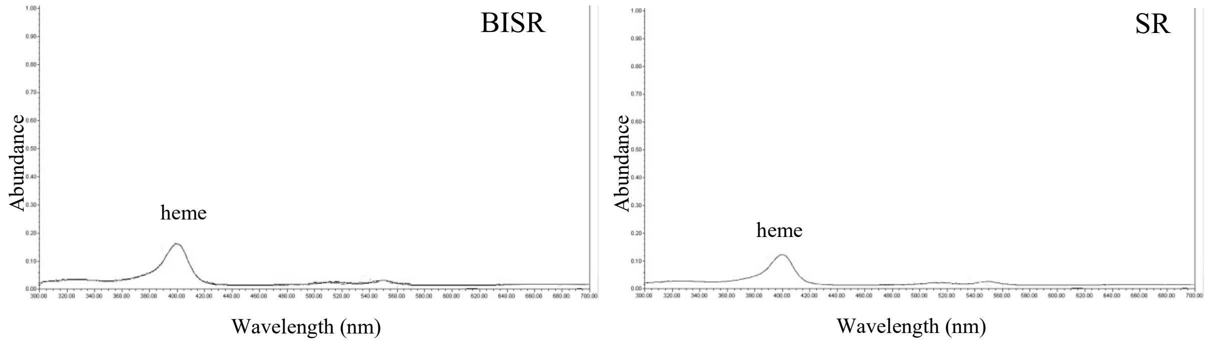

Figure S11. UV–Vis spectra of organic extracts of sediments of BISR and SR obtained using HPLC-PAD

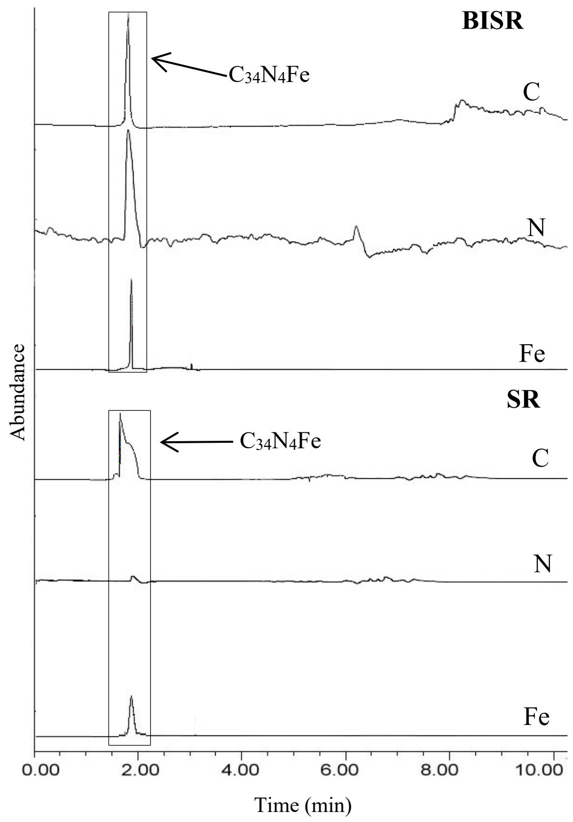

Figure S12. Detection of heme (carbon, nitrogen, and iron) in BISR and SR—GC-AED chromatograms. AED chromatogram of heme is presented in Figure S5 (Supplementary information A).
