# Supplementary material for: Sedimentary Cobalt Protoporphyrin as a Potential Precursor of Prosthetic Heme Group for Bacteria Inhabiting Fossil Organic Matter-Rich Shale Rock
Source: Biomolecules. 2021 Dec 20;11(12):1913. doi: 10.3390/biom11121913 (PMC8699415; doi:10.3390/biom11121913)
Supplement: Supplementary file 1 [file biomolecules-11-01913-s001.zip › Supplementary information/Supplementary information C. Supplementary results of the chemical analyses of the SR-BC and SR-SC samples.pdf]

Supplementary information C. Supplementary results of the chemical analyses of the SR-BC and SR-SC samples

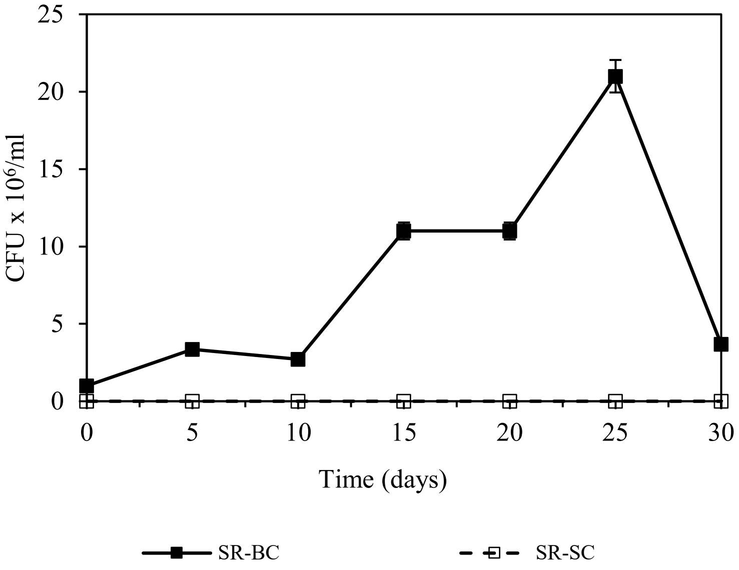

Figure S13. Growth curve of LM27 strain cultured on mineral medium with SR (SR-BC)

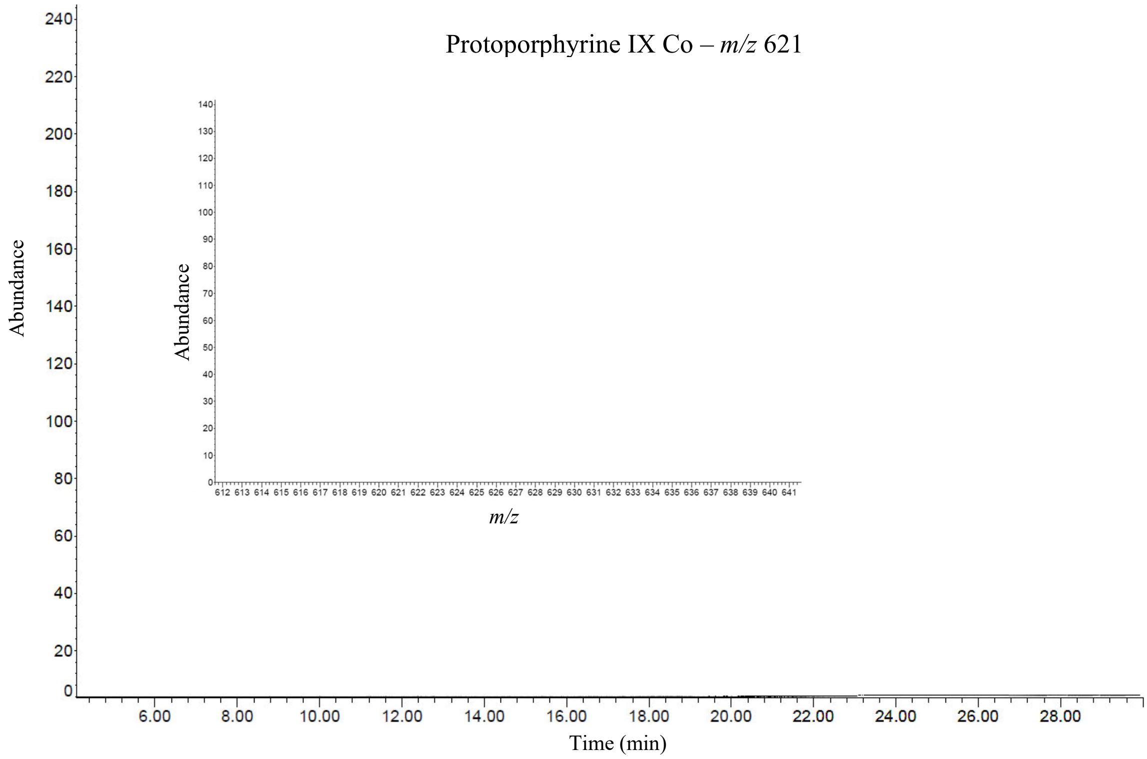

Figure S14. SIM chromatogram and mass spectrum (inset) of chloroform extract of the supernatant of SR-BC. SIM chromatogram of CoPPIX is presented in Figure S1 (Supplementary information A).

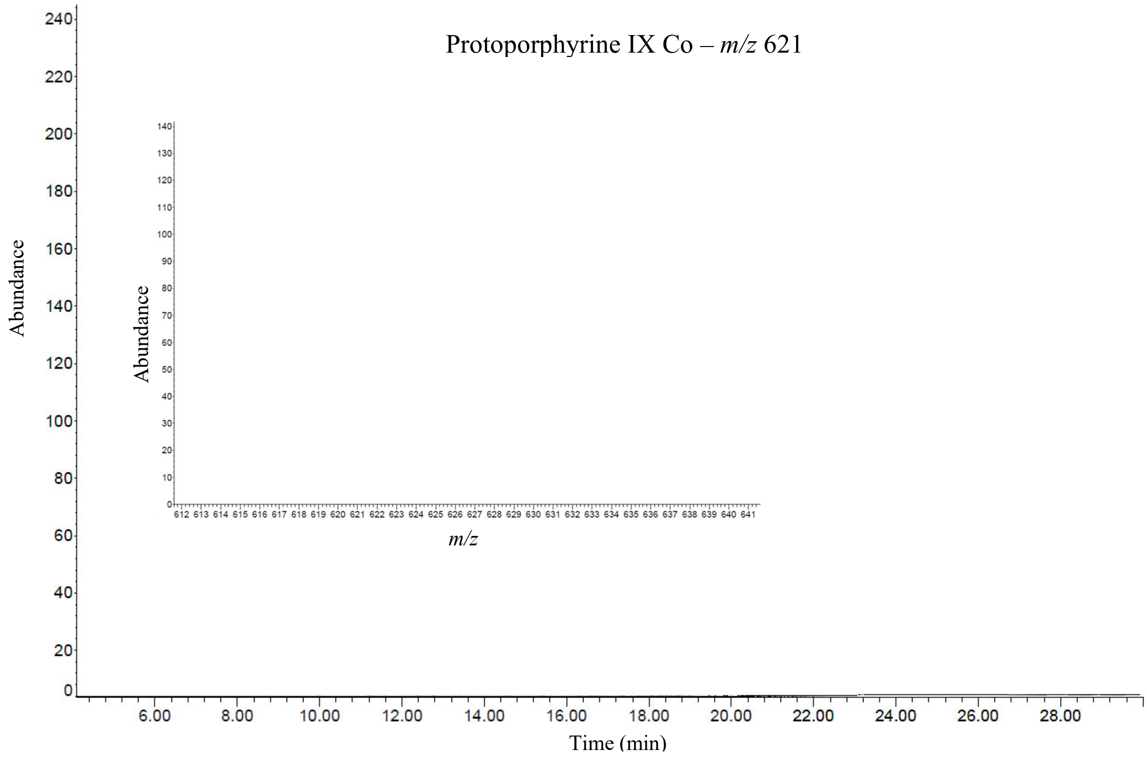

Figure S15. SIM chromatogram and mass spectrum (inset) of dichloromethane:methanol extract of the sediment of SR-BC. SIM chromatogram of CoPPIX is presented in Figure S1 (Supplementary information A)

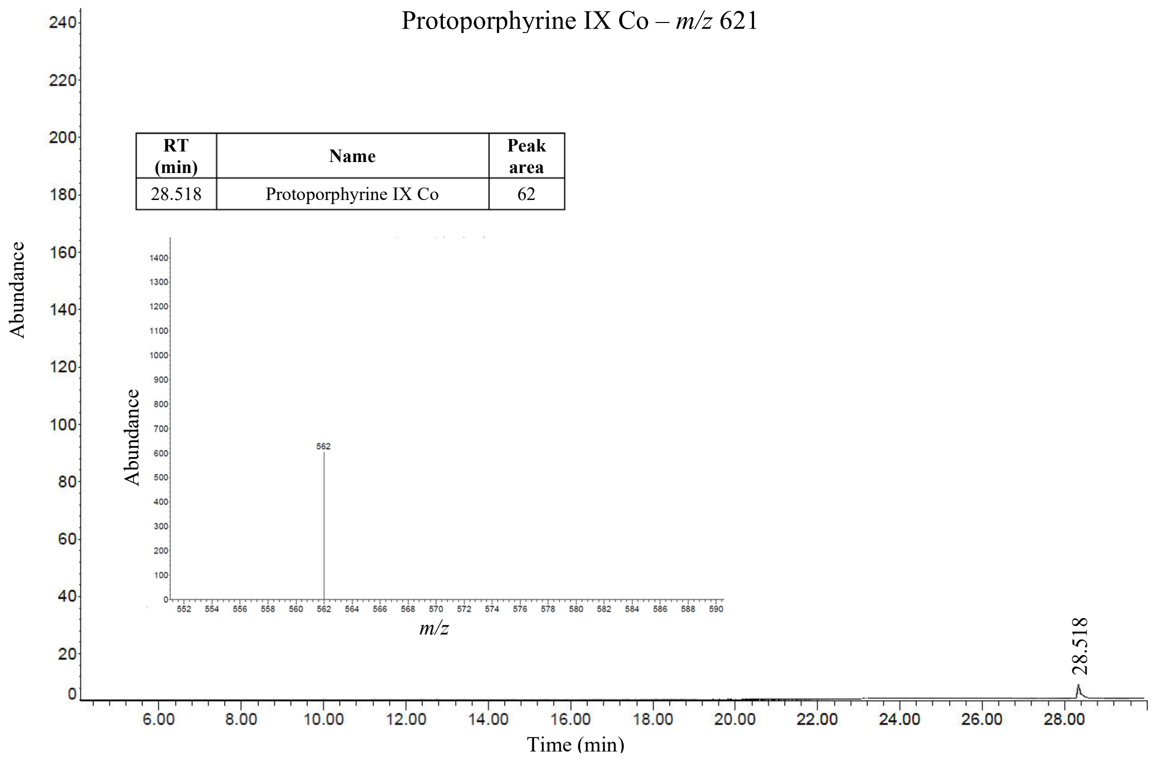

Figure S16. SIM chromatogram and mass spectrum (inset) of chloroform extract of the supernatant of SR-SC. SIM chromatogram of CoPPIX is presented in Figure S1 (Supplementary information A)

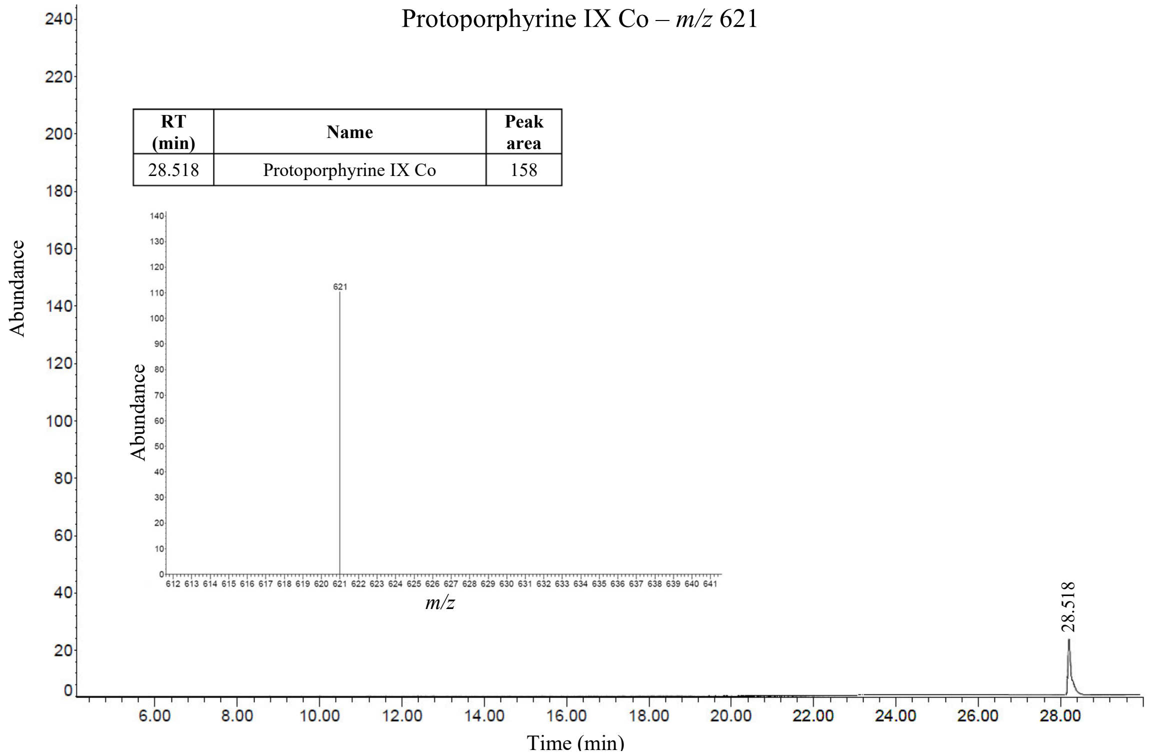

Figure S17. SIM chromatogram and mass spectrum (inset) of dichloromethane:methanol extract of the sediment of SR-SC. SIM chromatogram of CoPPIX is presented in S1 (Supplementary information A)

Table S2. Concentrations of CoPPIX in SR-BC and SR-SC based on GC-MS analyzes of *m/z* 621

Sample	Sample			SD	Average	Unit
	**1**	**2**	**3**			
SR-BC supernatant	0	0	0	0	0	µM
SR-BC sediment	0	0	0	0	0	µmol/kg
SR-SC supernatant	0.020	0.020	0.020	1,53E-04	0.020	µM
SR-SC sediment	0.521	0.523	0.516	3,87E-03	0.520	µmol/kg

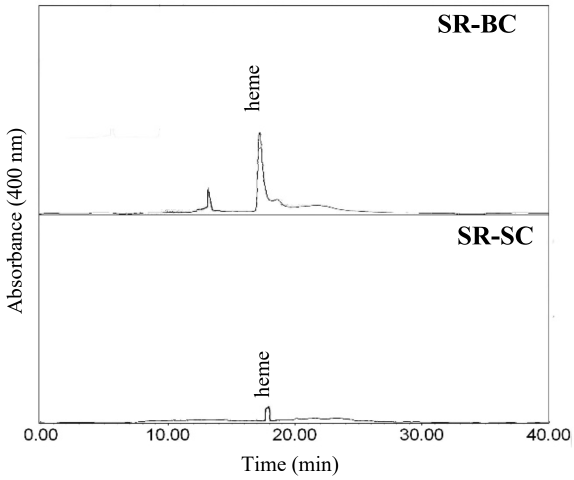

Figure S18. Detection of heme in SR-BC and SR-SC—HPLC-PAD chromatograms at 400 nm. HPLC-PAD chromatogram of heme is presented in Figure S3A (Supplementary information A)

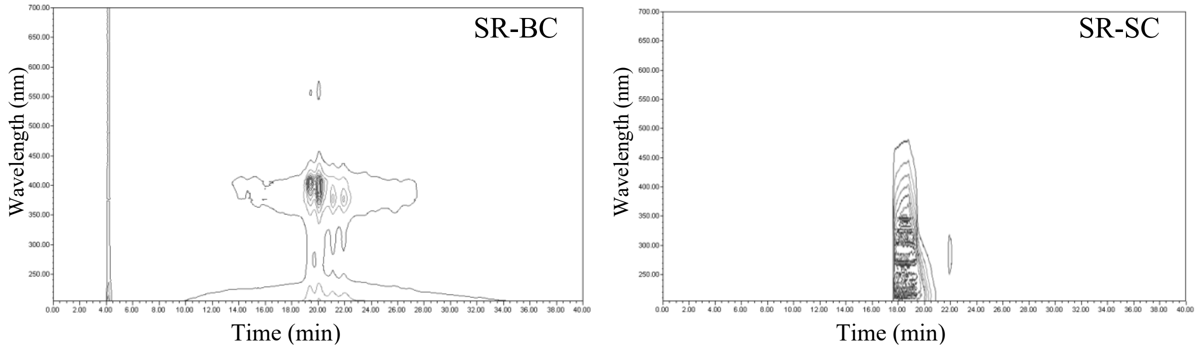

Figure S19. High-performance liquid 3D chromatograms of organic extracts of sediments of SR-BC and SR-SC obtained using HPLC-PAD

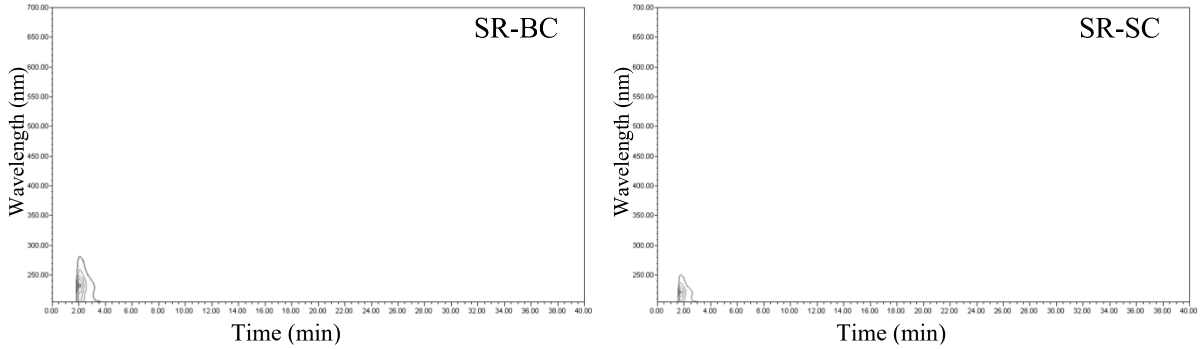

Figure S20. High-performance liquid 3D chromatograms of organic extracts of supernatants of SR-BC and SR-SC obtained using HPLC-PAD

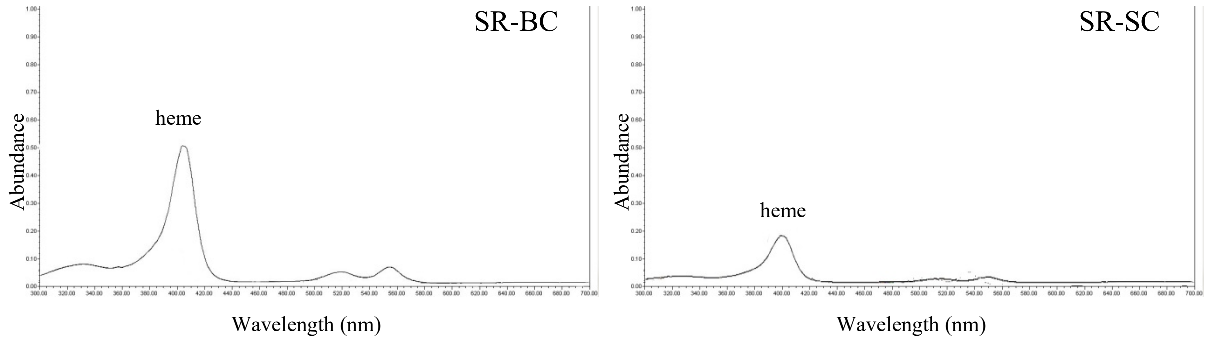

Figure S21. UV–Vis spectra of organic extracts of sediments of SR-BC and SR-SC obtained using HPLC-PAD

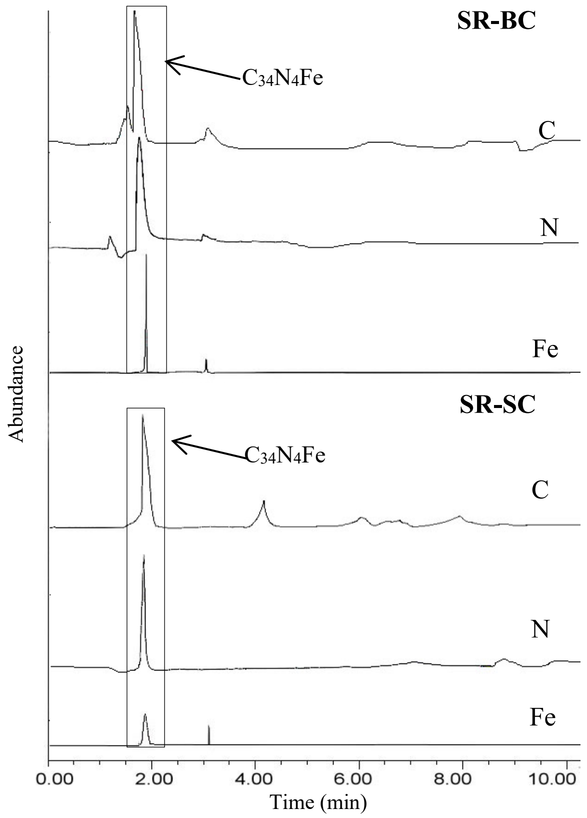

Figure S22. Detection of heme (carbon, nitrogen, and iron) in SR-BC and SR-SC—GC-AED chromatograms. AED chromatogram of heme is presented in Figure S5 (Supplementary information A)
