# Supplementary material for: Sedimentary Cobalt Protoporphyrin as a Potential Precursor of Prosthetic Heme Group for Bacteria Inhabiting Fossil Organic Matter-Rich Shale Rock
Source: Biomolecules. 2021 Dec 20;11(12):1913. doi: 10.3390/biom11121913 (PMC8699415; doi:10.3390/biom11121913)
Supplement: Supplementary file 1 [file biomolecules-11-01913-s001.zip › Supplementary information/Supplementary information D. Supplementary results of the chemical analyses of the CoPPIX-BC and CoPPIX-SC samples.pdf]

Supplementary information D. Supplementary results of the chemical analyses of the CoPPIX-BC and CoPPIX-SC samples

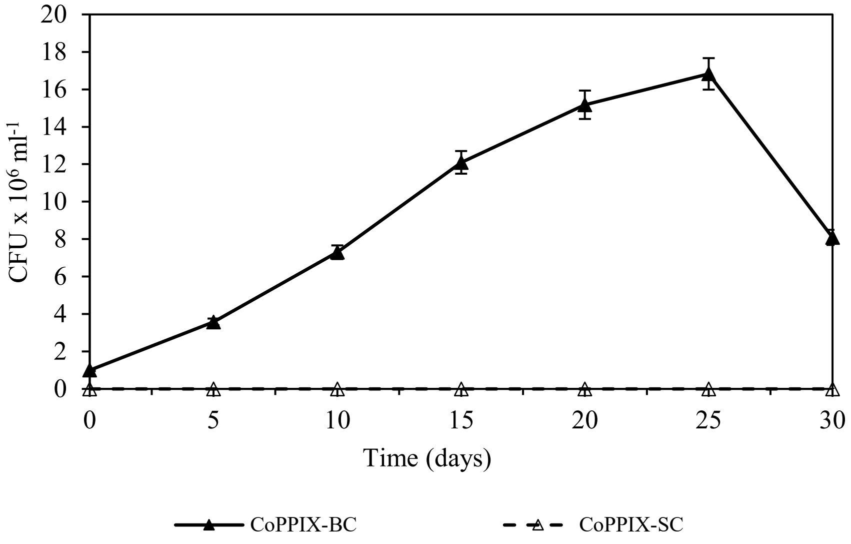

Figure S23. Growth curve of LM27 strain cultured on mineral medium with CoPPIX (CoPPIX-BC)

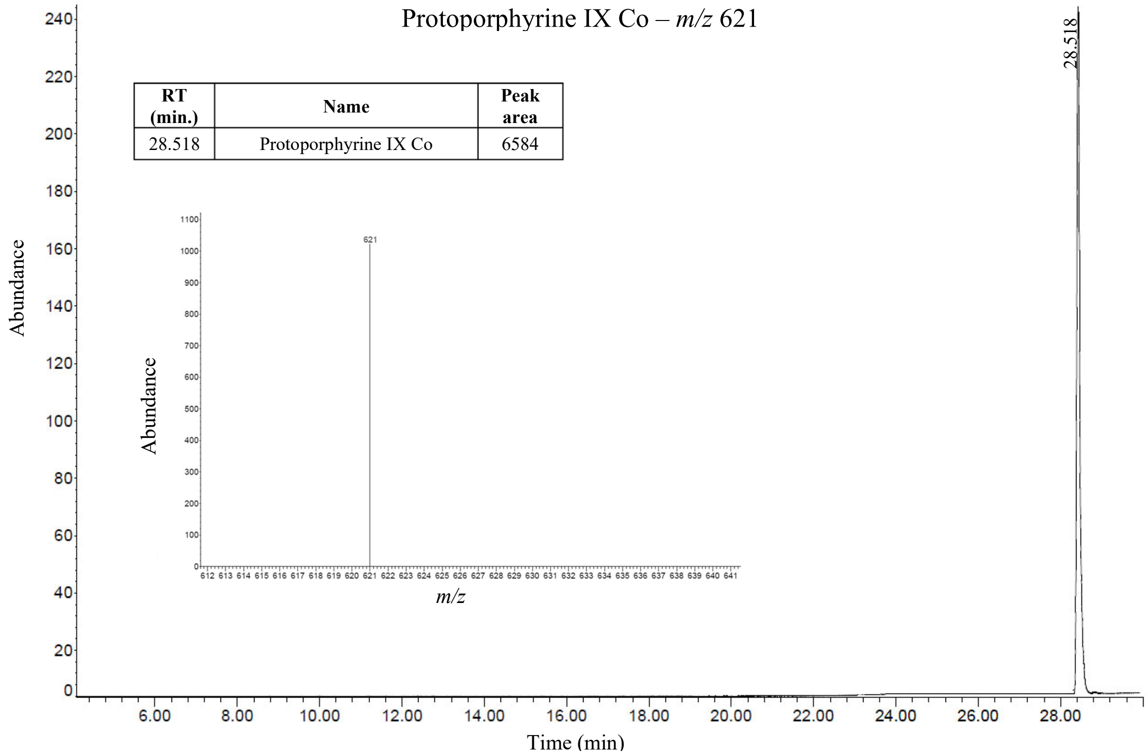

Figure S24. SIM chromatogram and mass spectrum (inset) of chloroform extract of the supernatant of CoPPIX-BC. SIM chromatogram of CoPPIX is presented in Figure S1 (Supplementary information A).

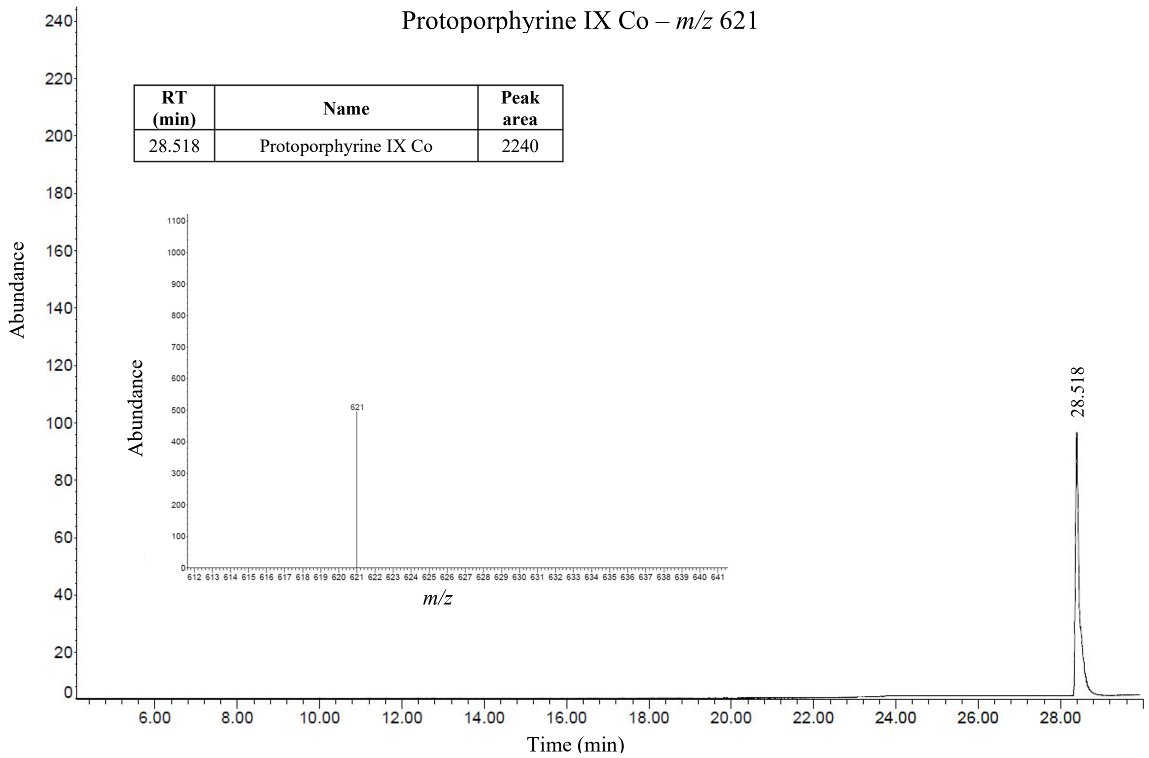

Figure S25. SIM chromatogram and mass spectrum (inset) of dichloromethane:methanol extract of the sediment of CoPPIX-BC. SIM chromatogram of CoPPIX is presented in Figure S1 (Supplementary information A).

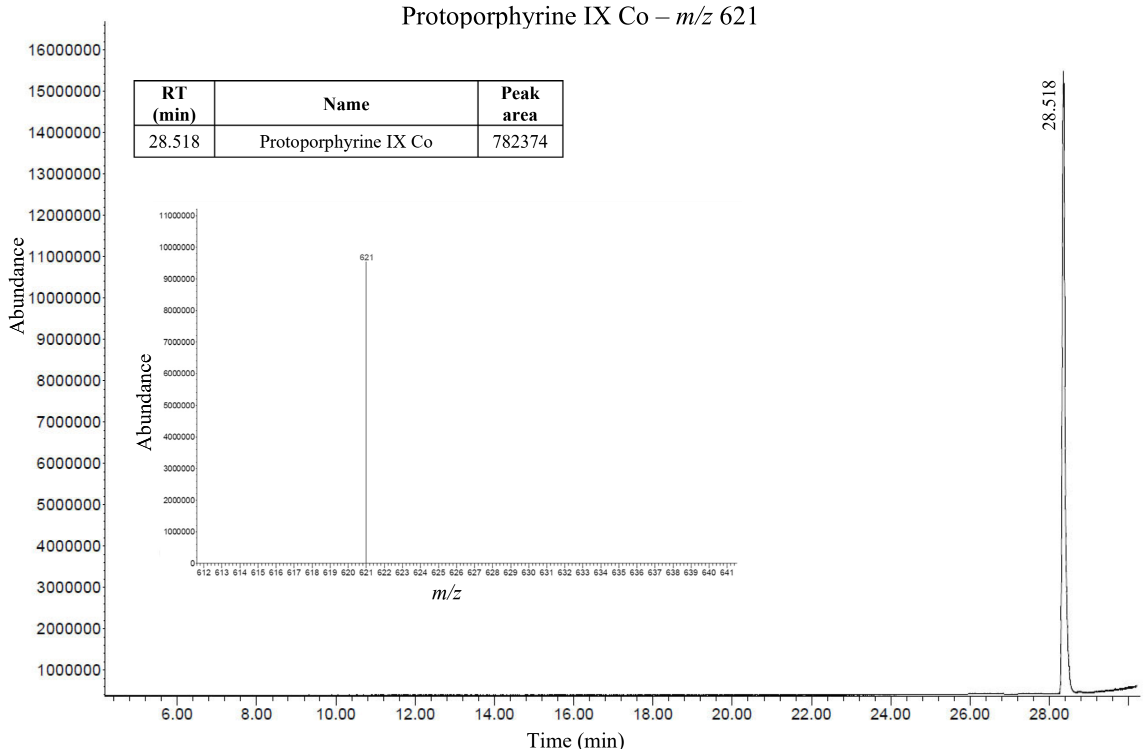

Figure S26. SIM chromatogram and mass spectrum (inset) of chloroform extract of the supernatant of CoPPIX-SC. SIM chromatogram of CoPPIX is presented in Figure S1 (Supplementary information A).

Table S3. Concentrations of CoPPIX in CoPPIX-BC and CoPPIX-SC based on GC-MS analyzes of *m/z* 621

**Sample**	**Sample**			**SD**	**Average**	**Unit**
	**1**	**2**	**3**			
PPIXCo-BC supernatant	2.175	2.173	2.176	1.6E-03	2.175	µM
PPIXCo-BC sediment	0.090	0.090	0.090	2.4E-05	0.090	µmol/g wet biomass
PPIXCo-SC supernatant	50.063	50.036	50.120	4.3E-02	50.073	µM
PPIXCo-BC sediment	0	0	0	0	0	-

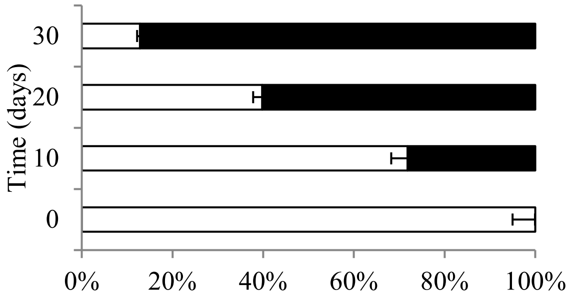

Figure S27. Accumulation of CoPPIX by LM27 strain: percentage content of cobalt in supernatant and cells of CoPPIX-BC after 10, 20 and 30 days of cultivation

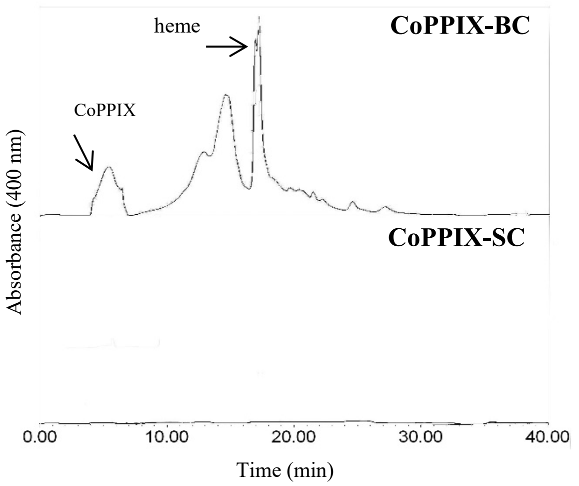

Figure S28. Detection of heme in the sediments of CoPPIX-BC and CoPPIX-SC—HPLC-PAD chromatograms at 400 nm. HPLC-PAD chromatogram of heme is presented in Figure S13A (Supplementary information A).

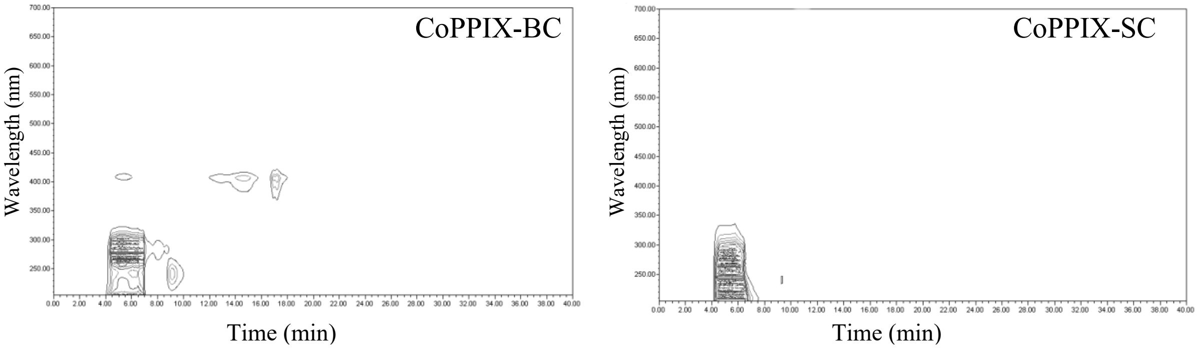

Figure S29. High-performance liquid 3D chromatograms of organic extracts of sediments obtained using HPLC-PAD

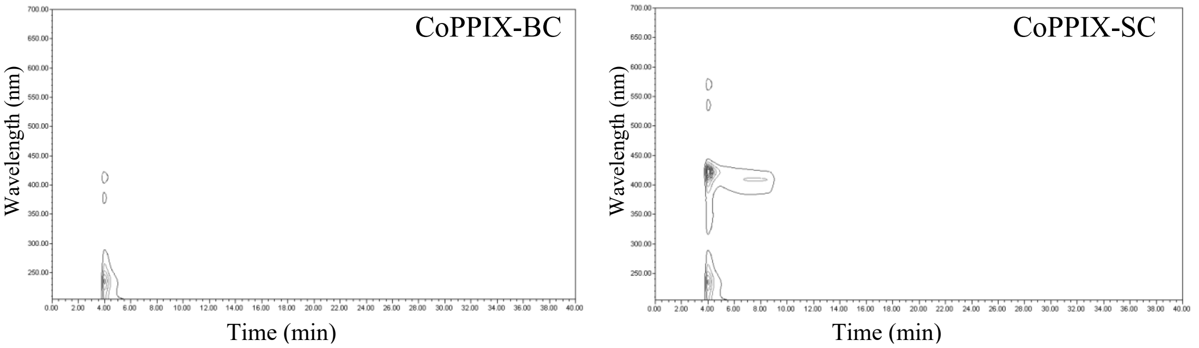

Figure S30. High-performance liquid 3D chromatograms of organic extracts of supernatants obtained using HPLC-PAD

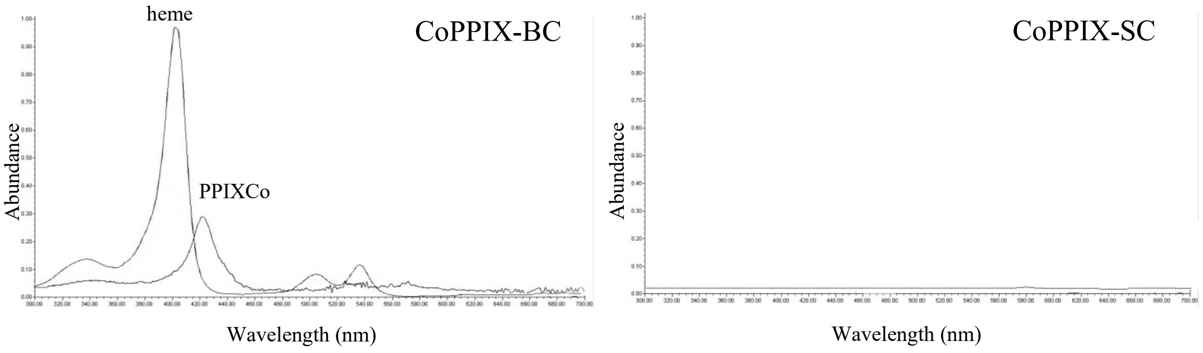

Figure S31. UV–Vis spectra of organic extracts of sediments obtained using HPLC-PAD

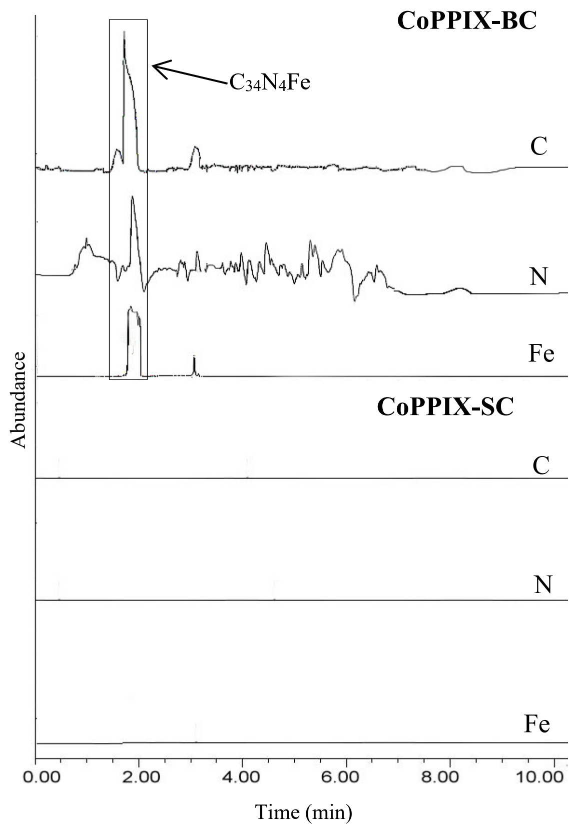

Figure S32. Detection of heme (carbon, nitrogen, and iron) in CoPPIX-BC and CoPPIX-SC—GC-AED chromatograms. AED chromatogram of heme is presented in Figure S5 (Supplementary information A).
