# Supplementary material for: Sedimentary Cobalt Protoporphyrin as a Potential Precursor of Prosthetic Heme Group for Bacteria Inhabiting Fossil Organic Matter-Rich Shale Rock
Source: Biomolecules. 2021 Dec 20;11(12):1913. doi: 10.3390/biom11121913 (PMC8699415; doi:10.3390/biom11121913)
Supplement: Supplementary file 1 [file biomolecules-11-01913-s001.zip › Supplementary information/Supplementary information E. Supplementary results of metaproteome (BISR) and proteomes (SR-BC, CoPPIX-BC) analyses.pdf]

Supplementary information E. Supplementary results of metaproteome (BISR) and proteomes (SR-BC, CoPPIX-BC) analyses

Table S4. Numbers of sequences of enzymes involved in heme biosynthesis and uptake and cobalt insertion/disconnection detected in the metaproteome (BISR) and proteomes (SR-BC, CoPPIX-BC); EC – enzyme cataloque

**Enzyme**	**Meta/proteome**		
	**BISR**	**SR-BC**	**PPIXCo-BC**
**Heme biosynthesis**			
5-aminolevulinate synthase EC: 2.3.1.37	-	-	-
porphobilinogen synthase EC: 4.2.1.24	gi491027513, WP_004889204.1, CAA35467.1, WP_004889204.1, WP_002458905.1	WP_171700908.1, NNU91633.1, WP_012574296.1, WP_077427980.1, WP_164970121.1, QAV27410.1, WP_064220470.1, WP_192953941.1, WP_091704482.1, WP_162829819.1, GAC90775.1, WP_043967315.1, MBA2878521.1, WP_181520405.1, WP_171334820.1, WP_183241280.1, WP_183249665.1, WP_081977254.1, KIQ93786.1, KFZ41698.1, WP_099668858.1, WP_141653475.1, WP_143255537.1, CUA78709.1, OSX54462.1, WP_003396409.1, WP_163150307.1, EPZ39665.1, EMI10609.1, WP_026011514.1 WP_035065471.1, WP_132947612.1, WP_183253443.1, WP_111644614.1, WP_044894615.1, WP_181537467.1, WP_066326516.1, WP_183183572.1, WP_027409488.1, WP_044742938.1, WP_080862119.1, WP_090947548.1, WP_159720222.1, WP_181554598.1, WP_043905655.1, WP_064549662.1, WP_154709999.1, WP_098305099.1, WP_035431564.1, WP_143415105.1, WP_015864684.1, WP_090918682.1, WP_012095637.1, WP_017435491.1, WP_097354818.1, WP_098914060.1, WP_090996694.1, WP_003248840.1, WP_042406500.1, WP_062753092.1, WP_089097970.1, WP_129727881.1, WP_013400298.1	GGE75935.1, WP_188389021.1, WP_020059980.1, WP_149178124.1, WP_174098839.1, WP_166910561.1, WP_097849105.1, WP_016092374.1, WP_098337914.1, WP_017150631.1, WP_087985328.1, WP_006096072.1
hydroxymethylbilane synthase EC: 2.5.1.61	AAA67601.1, WP_006599024.1, WP_011275555.1, EDN66896.1, WP_007473976.1, gi|493647429, WP_006599024.1, SEA14390.1, WP_038352255.1	WP_013379107.1, WP_090410885.1, RRF93330.1, WP_133964473.1	WP_118518172.1, WP_096920133.1, WP_074617978.1, WP_082669377.1, WP_014357354.1, WP_186841531.1, WP_070369550.1
uroporphyrinogen III synthase EC: 4.2.1.75	-	WP_104683532.1, WP_057456053.1, WP_122259343.1, WP_005781282.1, WP_002551372.1, WP_135003685.1, WP_122280121.1, WP_058974945.1	WP_058402446.1, WP_011167310.1, WP_134941604.1, WP_057433961.1, WP_044324127.1

uroporphyrinogen decarboxylase EC: 4.1.1.37	gi|652459094, WP_026853903.1, WP_002472704.1, WP_011275429.1	EFQ0483577.1, WP_024233173.1, WP_158120995.1	WP_046082250.1, WP_112044854.1, EGE0862999.1, HAI6559394.1, WP_032247939.1, WP_000137653.1, EEQ3568864.1, EFG5749303.1, WP_023308555.1, WP_130570441.1, WP_000137634.1, WP_072665359.1
coproporphyrinogen III oxidase EC: 1.3.3.3	OKQ85218.1	-	KJJ14657.1, OKR61434.1, OKQ85218.1, SQK83851.1, CDH74354.1
oxygen-independent coproporphyrinogen III oxidase EC: 1.3.98.3	gi659868130, WP_029915001.1, gi684178909, gbEAQ38129.3	WP_119522890.1	WP_113886989.1, WP_134302878.1, WP_171889427.1, WP_070339703.1, WP_023089009.1, WP_115759736.1, WP_070338550.1, WP_023518539.1, PXC07943.1, WP_160511252.1, WP_124141246.1, WP_181261645.1, WP_116830028.1, WP_126862502.1, WP_134310010.1, WP_089034942.1, WP_128538975.1
protoporphyrin/ coproporphyrin III oxidase EC: 1.3.3.4	-	-	-
menaquinone-dependent protoporphyrinogen III oxidase EC: 1.3.5.3	-	-	-
protoporphyrinogen IX oxidase EC: 1.3.99	-	-	-
protoporphyrin/coproporphyrin ferrochelatase EC: 4.99.1.1	WP_011275430.1, WP_027696985.1, WP_003099324.1, WP_021264089.1, AAT51432.1	WP_121784350.1, WP_031686597.1, WP_136310185.1, WP_174218239.1, WP_058202634.1, WP_094776821.1, WP_132457701.1	WP_126475982.1, WP_160518319.1, WP_033877308.1, WP_094776821.1, WP_132457701.1, WP_121784350.1, WP_031686597.1
**Heme uptake**			
heme acquisition protein HasA	-	-	-
periplasmic protein TonB	WP_197862675.1	WP_138407792, WP_138411937.1, WP_148924790.1, WP_181112699.1, WP_152489604.1, WP_102834729.1, WP_138486174.1	WP_148924790.1, WP_181112699.1, WP_152489604.1, WP_102834729.1

hemoglobin/transferrin/lactoferrin receptor protein (hemr)	WP_058169809.1, WP_182150463.1, WP_128672018.1, WP_003095535.1, WP_022580007.1	WP_128672018.1, WP_003095535.1, WP_022580007.1, WP_058159931.1, WP_033990026.1, WP_132444818.1, WP_003135463.1, WP_134572733.1, WP_134633887.1, WP_009314330.1, WP_033954696.1, WP_058150557.1, WP_034021499.1, WP_132868020.1, WP_116820038.1, WP_033963567.1, WP_024947020.1, WP_121382417.1, WP_034036579.1, WP_132497680.1, WP_126896166.1, WP_124988305.1, WP_126861255.1, WP_096069494.1	WP_058169809.1, WP_182150463.1, WP_124133528.1, WP_125909932.1, WP_033990026.1, WP_132444818.1, WP_003135463.1
biopolymer transport protein ExbB	WP_164634537.1	WP_197962956.1, WP_152102413.1, TAI63057.1, WP_158669561.1, WP_028140254.1, WP_194471296.1, WP_027278412.1, WP_066509674.1, OCX26818.1, WP_094194199.1, WP_035998843.1, OIM94023.1, WP_100952068.1, WP_152977387.1	WP_022724321.1, WP_107346470.1, WP_107357418.1, WP_142882041.1, WP_041798587.1, ABE41223.1, WP_182746610.1, WP_092686109.1, MBI5130876.1, WP_054162619.1, WP_114357654.1, OPF91494.1, WP_110788226.1, WP_119855070.1, NVN85044.1, WP_089025497.1, WP_139486359.1, VIO80036.1, WP_139864777.1, WP_106326951.1, WP_194471296.1, WP_027278412.1, WP_066509674.1, OCX26818.1, WP_094194199.1, WP_035998843.1, OIM94023.1, WP_100952068.1, WP_152977387.1, WP_036041101.1, WP_095736165.1, RZN28968.1
biopolymer transport protein ExbD	WP_072840788.1	WP_012071749.1, WP_111608707.1, WP_113918029.1, WP_112135015.1, WP_176336467.1, WP_111639718.1, WP_024025681.1, WP_123095856.1	WP_113918029.1
**Cobalt insertion/disconnection**			
sirohydrochlorin cobaltochelatase/sirohydrochlorin cobalt-lyase EC: 4.99.1.3	WP_060613678.1, WP_100263700.1, WP_149632279.1, WP_042956680.1, WP_027912619.1, WP_028237679.1, WP_024616339.1, WP_092412700.1, WP_114731145.1	WP_003199795.1, WP_053183376.1, WP_092462015.1, WP_039011076.1, WP_043041964.1, WP_186728245.1, WP_053120776.1	WP_092145369.1, WP_063322157.1, WP_043041964.1, WP_186728245.1, WP_092223853.1, WP_053178647.1

Table S5. Numbers of hemoprotein sequences detected in the metaproteome (BISR) and proteomes (SR-BC, CoPPIX-BC)

**Hemoprotein**	**Meta/proteome**		
	**BISR**	**SR-BC**	**CoPPIX-BC**
Cytochromes	gi|499245897, gi|493924522, gi|330949201, gi|268584477, gi|499630345, gi|498185039, gi|499630342, gi|517333069, gi|519012058, gi|488713780, gi|490102597, gi|671493207, gi|631847638, gi|497318490, gi|519020998, gi|353255924, gi|504263092, gi|124514988, gi|344169056, gi|503795255, gi|504774832, gi|503795252, gi|495086913, gi|124515016, gi|320462, gi|329112144, gi|499595441, gi|653251020, gi|499595357, gi|494666478, gi|410732281, gi|76667604, gi|543962213, gi|302039157	gi|504667349, gi|489380605, gi|739667945, gi|648288579, EKG43499.1, gi|516498201, gi|392421384, gi|146284245, gi|489379413, gi|739176448	gi|602235550, gi|950761959, gi|1027764433, gi|545655965, gi|951276957, gi|521092032, gi|737997068, Q8KS19, gi|489068679, gi|501226912, gi|653577710, gi|489376117, gi|489376656, gi|489380605, gi|739667945, gi|648288579, gi|516498201, gi|392421384, gi|146284245, P81154, Q52369, gi|674642377, C1DN39, Q9I3M9, gi|739176448, gi|26988052, gi|489379413, gi|498175705, gi|504667349, gi|492990377, gi|737247626, gi|21702709
Catalases	gi|494666045, KXK49644.1, gi|1698550, gi|504938131, gi|491324047, gi|3927890, gi|520401, gi|500251659, gi|504938131, gi|499595129	gi|500260364, PYN24457.1, KPX01798.1, gi|516497823, CJL05199.1	gi|516497823, Q9I1W8, gi|500260364, A4VK13, B0TRN5
Peroxidases	gi|489375670, gi|515815228, gi|488371807, gi|503795169, gi|498325609	gi|692344295, KFJ89559.1, gi|489377300	gi|489378348, gi|692344295, gi|489377300, gi|514327837, gi|495264620
